# *Ilex paraguariensis* (Yerba Mate): A Narrative Review of Bioactive Compounds, Biological Activities, and Green Extraction Technologies

**DOI:** 10.3390/nu18152535

**Published:** 2026-08-04

**Authors:** Farhang Hameed Awlqadr, Syamand Ahmed Qadir, Abdulrahman H. Laftah, Othman Abdulrahman Mohammed, Tablo H. Salih, Mohammed N. Saeed, Waleed Al Abdulmonem, Noor Alriyahi, Ammar Badran Ramddan, Hassan Raza

**Affiliations:** 1 Food Science and Quality Control, Halabja Technical College, Sulaimani Polytechnic University, Sulaymaniyah 46001, Iraq; farhang.hamid.a@spu.edu.iq; 2 Medical Laboratory Techniques Department, Halabja Technical Institute, Research Center, Sulaimani Polytechnic University, Sulaymaniyah 46001, Iraq; syamand.qadir@spu.edu.iq (S.A.Q.); tablo.salih.tcas@spu.edu.iq (T.H.S.); 3 Food Science Department, College of Agriculture, University of Basrah, Basrah 61004, Iraq; abdulrahman.laftah@uobasrah.edu.iq; 4 Medical Laboratory Science Department, Halabja Technical College, Sulaimani Polytechnic University, Sulaymaniyah 46001, Iraq; othman.mohammed@spu.edu.iq; 5 Department of Nutritional Analysis and Health, Kifri Technical College, Garmian Polytechnic University, Sulaimaniyah 46001, Iraq; mohammed.noori@gpu.edu.iq; 6 Department of Pathology, College of Medicine, Qassim University, Buraidah 51411, Saudi Arabia; 7 Anesthesia Techniques Department, College of Health and Medical Technologies, Al-Farqadein University, Basrah 61004, Iraq; noor.adbulameer@fu.edu.iq; 8 Faculty of Food Science and Nutrition, Bahauddin Zakariya University, Multan 60800, Pakistan; sh.raza40@yahoo.com

**Keywords:** *Ilex paraguariensis*, yerba mate, metabolic health, inflammation, antimicrobial activity, clinical evidence, extraction standardization, safety

## Abstract

Background: *Ilex paraguariensis* (yerba mate) is a widely consumed botanical beverage rich in caffeoylquinic acids, methylxanthines, flavonoids, and saponins. However, the large number of biological effects attributed to yerba mate has made it difficult to distinguish mechanistic promise from clinically relevant evidence. Methods: This structured narrative review searched PubMed/MEDLINE, Scopus, Web of Science Core Collection, ScienceDirect, Wiley Online Library, and Google Scholar for literature published from January 2015 through June 2026. Predefined thematic search blocks, eligibility criteria, staged screening, data extraction, and design-appropriate methodological appraisal were applied. Results: The most coherent evidence concerns antioxidant and anti-inflammatory mechanisms and possible metabolic effects, including lipid, glycemic, and body-weight-related outcomes. However, much of the evidence remains preclinical, while human studies are limited, heterogeneous in dose and formulation, and generally of short duration. Safety depends on total caffeine exposure, preparation temperature, processing-related contaminants, individual susceptibility, and concurrent medication use. Very-hot consumption and smoke-derived polycyclic aromatic hydrocarbons represent avoidable risks. Conclusions: Yerba mate may represent a functional beverage and source of bioactive ingredients, but therapeutic claims remain unsupported. Responsible use requires control of caffeine dose, beverage temperature, processing contaminants, and exposure in vulnerable populations. Future research should prioritize standardized preparations, long-term safety assessment, dose–response characterization, and adequately powered clinical trials.

## 1. Introduction

Yerba mate has attracted scientific interest not only as a caffeinated beverage but also as a source of polyphenols, methylxanthines, flavonoids, and saponins that may influence oxidative, inflammatory, and metabolic pathways [1,2]. Chlorogenic, caffeic, and neochlorogenic acids, together with caffeine, theobromine, and rutin, have demonstrated antioxidant, anti-inflammatory, lipid-related, and antimutagenic activities in chemical assays, cell systems, and animal models [3,4]. These findings establish biological plausibility but should not be interpreted as evidence of clinical efficacy.

A limited number of human studies have reported changes in lipid profiles, body-weight-related measurements, or glycemic biomarkers following yerba mate consumption [5,6]. However, these studies are generally limited by small samples, short intervention periods, heterogeneous preparations, inconsistent doses, and incomplete control of dietary and lifestyle factors. Consequently, the available evidence does not establish yerba mate as a treatment or preventive intervention for obesity, diabetes, cardiovascular disease, or other chronic disorders.

In addition to limitations in clinical study design, differences in processing, extraction, and formulation are important sources of heterogeneity among yerba mate studies. Advanced extraction approaches, including ultrasound-assisted extraction (UAE), microwave-assisted extraction (MAE), and green-solvent systems, may improve the recovery, stability, and standardization of bioactive compounds for use in functional foods and nutraceutical formulations [2]. Interest in plant-derived functional ingredients is also increasing in the Middle East, where traditional medicinal plant use and the high burden of non-communicable diseases may encourage further investigation of yerba mate; however, its clinical relevance remains to be established [1,7]. Its potential cultivation under dry or semi-dry conditions and its integration into sustainable production systems require region-specific agronomic validation [8]. Continued advances in extraction and formulation may support the future development of standardized yerba-mate-derived food and nutraceutical products [9].

Particular attention is given to (i) the bioactive constituents most plausibly linked to biological effects, (ii) the strength and limitations of evidence for antioxidant, anti-inflammatory, and metabolic outcomes, (iii) the extent to which neuroprotective, antimicrobial, and anticancer findings remain preclinical, (iv) the influence of extraction and formulation on chemical standardization and reproducibility, and (v) safety considerations related to dose, caffeine exposure, preparation temperature, processing contaminants, vulnerable populations, medication interactions, and regulatory quality control.

## 2. Search Methodology

### 2.1. Review Design and Scope

This review was conducted as a structured narrative review to critically identify, compare, and synthesize evidence concerning the botanical and raw-material characteristics, phytochemical and nutritional composition, extraction-dependent variability, pharmacological activities, safety, and potential food and nutraceutical applications of *Ilex paraguariensis* (yerba mate). The review was not designed as a systematic review or meta-analysis, and no quantitative pooling was planned because the eligible evidence encompassed heterogeneous analytical, extraction optimization, in vitro, animal, observational, and intervention studies. Nevertheless, a predefined framework for literature identification, eligibility assessment, study selection, data extraction, methodological appraisal, and evidence synthesis was applied to improve transparency, reproducibility, and consistency of interpretation.

### 2.2. Information Sources and Search Period

The literature search covered publications from January 2015 through June 2025 and was updated in June 2025. PubMed/MEDLINE, Scopus, and Web of Science Core Collection were used as the principal bibliographic databases. ScienceDirect and Wiley Online Library were searched as publisher platforms, and Google Scholar was used as a supplementary scholarly search engine to identify additional relevant records. Reference lists of eligible primary studies, systematic reviews, and meta-analyses were examined manually to identify studies not retrieved by the electronic searches. Publications predating 2015 were considered only when they represented essential landmark evidence on taxonomy, traditional use, chemical characterization, analytical methodology, or biological mechanisms that could not be adequately replaced by a more recent source.

### 2.3. Search Strategy

Search terms were adapted to the indexing rules and syntax of each information source. The complete database-specific strategies, including controlled vocabulary, free-text terms, field codes, and applied limits, are provided in Supplementary Material S1. Additional searches combined the species block with terms related to leaves, stems, roots, fruits, seeds, processing residues, morphology, genotype, functional foods, nutraceuticals, formulation, and standardization. In PubMed, searches were conducted using title/abstract terms and relevant indexed vocabulary where available. In Scopus, the terms were applied to title, abstract, and keyword fields; in Web of Science, they were applied as topic searches. Equivalent title, abstract, keyword, and full-text options were used in ScienceDirect, Wiley Online Library, and Google Scholar according to the functions available on each platform. Boolean operators (AND and OR), quotation marks, truncation symbols, and date and language filters were used where supported. Google Scholar was used to complement, rather than replace, the database searches, and potentially relevant records were checked against the predefined eligibility criteria before inclusion.

### 2.4. Eligibility Criteria and Study Selection

Studies were included when they investigated *Ilex paraguariensis* or yerba mate preparations and reported relevant data on composition, extraction, biological activity, health effects, safety, or food and nutraceutical applications. Eligible sources included peer-reviewed analytical, in vitro, animal, observational, clinical, systematic review, and meta-analytic studies available in English with sufficient methodological detail. Studies were excluded when they focused on other *Ilex* species, lacked extractable evidence, provided insufficient methodological information, or were outside the review scope. Conference abstracts, editorials, theses, patents, book chapters, non-peer-reviewed publications, and unavailable full texts were also excluded. For duplicate reports from the same study, the most complete publication was retained.

### 2.5. Screening and Study Selection

The database searches identified 2409 records. All retrieved records were imported into the reference management software EndNote 2025 and Zotero 7.0.32, where they were consolidated and screened for duplication based on titles, authors, publication years, journal names, DOIs, and PubMed identifiers. Following the removal of 1091 duplicate records, 1318 unique records remained for title and abstract screening. Potentially relevant articles were subsequently retrieved and assessed in full text according to the predefined eligibility criteria. Records were excluded because of insufficient relevance to *Ilex paraguariensis*, absence of extractable evidence, inadequate methodological reporting, ineligible publication type, or inability to distinguish the specific effects of yerba mate from those of other ingredients. After completion of the screening and eligibility assessment process, 1193 records were excluded, and 125 sources were retained for inclusion in the final narrative synthesis.

### 2.6. Data Extraction, Methodological Appraisal, and Evidence Synthesis

For studies meeting the eligibility criteria, relevant data were extracted on study design, plant origin and part, processing and extraction conditions, analytical methods, experimental model or participant characteristics, dose, duration, principal outcomes, and reported limitations. The findings were then organized into thematic categories covering botanical and compositional characteristics, extraction technologies, biological activities, human evidence, safety, and food or nutraceutical applications. Methodological quality was assessed using criteria appropriate to each study design. Human studies were evaluated for participant selection, intervention standardization, comparators, confounding, attrition, outcome validity, and statistical analysis. Animal and in vitro studies were examined for model relevance, extract characterization, dose selection, controls, replication, and assay validity, whereas analytical and extraction studies were assessed for botanical authentication, method validation, extraction parameters, precision, recovery, and use of suitable reference standards. The synthesis distinguished mechanistic and preclinical findings from demonstrated human health effects. Greater weight was assigned to consistent evidence from well-designed human studies, systematic reviews, and validated analytical investigations. Findings from poorly characterized extracts, high experimental doses, small samples, inadequate controls, or short follow-up periods were interpreted cautiously, and conflicting results were retained and critically discussed.

## 3. Botanical, Historical, and Raw-Material Context

### 3.1. Historical and Geographical Context

*Ilex paraguariensis* A. St.-Hil. (yerba mate) is a dioecious evergreen tree of the Aquifoliaceae family native to subtropical South America. Traditionally consumed by the Guaraní and Tupí peoples as an infusion known as *ka’a*, yerba mate later became embedded in regional culture through beverages such as *chimarrão* and *tereré* and has gained wider international interest because of its bioactive composition and potential functional properties [10].

### 3.2. Botanical and Anatomical Characteristics

**Figure 1:**
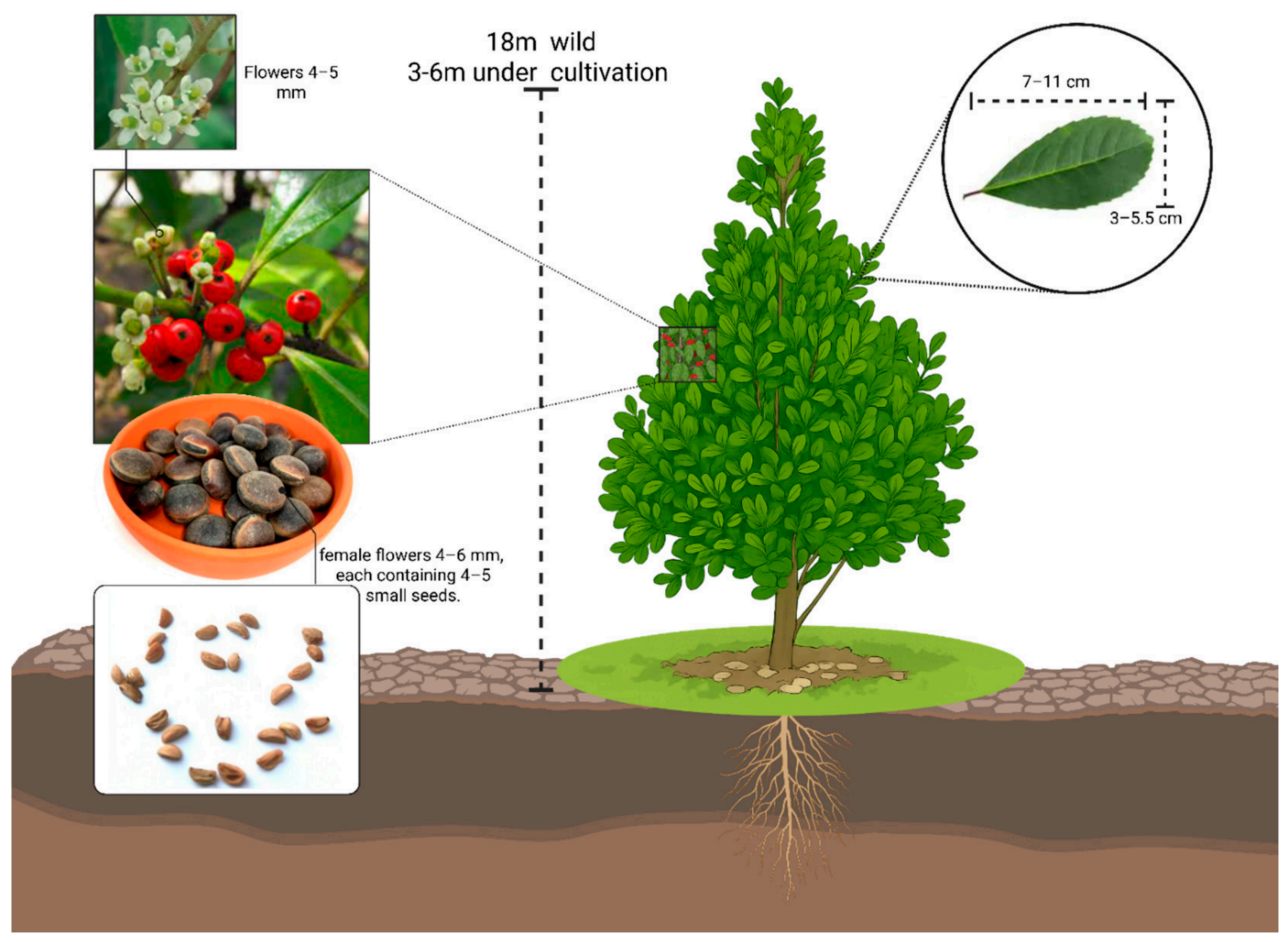
*Ilex paraguariensis* (yerba mate) showing tree and root form, leaves (7–11 cm × 3–5.5 cm), flowers, red berries, and seeds (4–5 seeds per fruit). Created in BioRender. Awlqadr, F. (2025) https://BioRender.com/9so5enq.

Under natural conditions, *Ilex paraguariensis* may reach approximately 18 m, although cultivated plants are typically maintained at 3–6 m to facilitate harvesting. It has a cylindrical grayish trunk and a deep taproot with fibrous lateral roots adapted to the acidic soils of the Atlantic Forest region [11,12]. The leaves are simple, alternate, evergreen, and coriaceous, usually measuring 7–12 × 3–5 cm, with a dark green adaxial surface, paler abaxial surface, serrate margins, and pinnate venation. Anatomically, the dorsiventral, hypostomatic lamina contains predominantly anomocytic stomata on the abaxial epidermis, a biconvex midrib with a collateral vascular bundle, and calcium oxalate crystals. These features support botanical identification and pharmacognostic quality control, while the leaves serve as the principal source of methylxanthines, caffeoylquinic acids, flavonoids, and saponins [13,14,15]. The species is dioecious, producing small white-to-greenish flowers in axillary cymes and red berries containing four to five seeds, as summarized in Figure 1.

### 3.3. Raw-Material Variability Relevant to Bioactivity

The leaves and young stems constitute the principal consumed and extracted matrices of yerba mate. However, their chemical composition varies according to genotype, cultivation environment, plant maturity, harvesting conditions, drying, roasting, and storage. These sources of variability should be considered when comparing pharmacological and nutritional studies, because apparently similar “yerba mate extracts” may contain markedly different concentrations of caffeoylquinic acids, caffeine, theobromine, flavonoids, and saponins [16].

The reproductive characteristics of the species contribute to the genetic diversity observed in wild populations and provide opportunities for selective breeding programs aimed at improving flower or seed production, yield, phytochemical content, and drought tolerance [17]. Yerba mate is cultivated in monoculture systems, forest understories, and agroforestry systems. Agroecological production systems, such as those developed in Misiones Province, Argentina, may improve resilience, preserve biodiversity, and support soil health through the cultivation of yerba mate within multispecies systems [18].

Therefore, studies investigating the chemical composition, extraction efficiency, or biological activity of yerba mate should clearly report the genotype or origin of the plant material, the plant part used, cultivation conditions, maturity stage, and postharvest processing procedures. These factors are essential for comparing findings among studies and for developing reproducible and standardized yerba mate extracts.

## 4. Bioactive Composition and Nutritional Relevance

*Ilex paraguariensis* is a plant species well-reputed for its broad phytochemical profile as well as its remarkable health-promoting properties. Its chemical constitution is composed of a variety of bioactive substances, minerals, macronutrients, sugars, organic acids, and fatty acids. These compounds are largely responsible for the potent antioxidant, anti-inflammatory, metabolic-regulatory, and antimicrobial activities of this beverage, turning it into a traditional drink and a potential functional food. The variable composition of *Ilex paraguariensis* contains a large number of bioactive compounds, minerals, fatty acids, macronutrients, and hydrophilic substances. Major phenolic acids, flavonoids, and methylxanthines are shown in Table 1.

Methylxanthines, phenolic acids, and flavonoids are among the most important bioactive compounds in yerba mate. The most abundant methylxanthine is caffeine (8.7–17.5 mg/g dry weight). It is a central nervous system stimulant that boosts alertness, mental performance, and metabolism. Theobromine, in smaller quantities (0.5–1.5 mg/g), gives mild diuretic and vasodilatation effects. Both substances contribute to the metabolism of energy and improvement of physical and sports performance [19].

Phenolic acids are also abundant, of which the predominant ones were chlorogenic acid (28.2–44.5 mg/g) and 5-caffeoylshikimic acid (43–47 mg/g). These compounds have strong antioxidant, antioxidative stress, hepatoprotective, and hypoglycemic activity [2,20]. Other phenolic acids, including neochlorogenic acid, isochlorogenic acid, caffeic acid, gallic acid, ferulic acid, and “3,4-*O*-dicaffeoylquinic acid”, also exhibit anti-inflammatory, antimicrobial, and anticancer activities [21]. Flavonoids from yerba mate have demonstrated antioxidant, inflammatory, and vascular-related activities primarily in experimental systems. These compounds significantly contribute to the anti-inflammatory and antioxidant activity of the plant [22]. Other unmeasured, but pharmacologically relevant compounds, such as rosmarinic acid, cyanidin (an anthocyanin), and caffeic acid phenethyl ester, confer pharmacological properties to the plant extract, including antimicrobial, neuroprotective, and anti-obesity activities. The mineral composition of yerba mate is also valuable and can be considered an important source of dietary essential elements. It is also rich in potassium (860–1050 mg/100 g), which is important for healthy heart and muscle function. It contains calcium (220–500 mg/100 g) and magnesium (200–350 mg/100 g), both with marquee functions for bone health, muscle contraction, and nerve conduction [23,24]. Oxygen transport, defense against disease, and energy generation in cells are enhanced by iron (2.2–7.8 mg/100 g), zinc (1.3–2.5 mg/100 g), and phosphorus (60–120 mg/100 g).

Trace minerals, including manganese, copper, and selenium, are also important for antioxidant enzyme systems and endocrine function. Although elements such as arsenic and its derivatives (As(III), As(V), DMA) are present as trace elements, it has been verified that their quantity does not exceed the standard amounts, and it is more dependent on cultivation and production technologies [24]. The fatty acid profile of yerba mate is also important in terms of its nutrition and functionality. The omega-3 type of polyunsaturated fatty acids (PUFAs), so called linolenic acid (C18:3n3), which was established as the dominant constituent (40 to 60.3%) of the profile, promotes to heart, anti-inflammatory, and brain functions [25]. Cardiovascular indices are also boosted by linoleic acid (C18:2n6), an omega-6 PUFA, and oleic acid (C18:1n9), a monounsaturated fatty acid. Saturated fatty acids, including palmitic acid (20.7–25.2%), stearic acid (2.6–4.6%), and myristic acid (0.96–1.4%), are involved in cell membrane formation and energy storage. Most interestingly, nervonic acid (0.97–1.7%), a monounsaturated fatty acid implicated in myelin and brain growth, is a central component these days. In addition to micronutrients, yerba mate also gives us a wealth of macronutrients. Nutrient levels vary throughout the whole plant, leaves, and stems. Leaves are more nutritious, containing the highest levels of ash (6.6%), proteins (26.1%), and fat (4.9%). Stems have lower levels of protein (20.8%) and fat (1.7%) and higher carbohydrates (73%), representing structural storage rather than bioactivity. Average energy values are nearly similar to all organs of the plant, varying from 1637 kJ/100 g dry weight to 1668 kJ/100 g dry weight. The caloric contribution would then be constant according to the location of the part of the plant. Yerba mate’s hydrophilic compound profile (sugars and organic acids) may compensate for its macronutrient richness and influence flavor and functionality. All parts are high in sucrose, especially the leaves (4.85 g/100 g), with moderate levels of glucose and fructose. Stems have a little more fructose (0.67 g/100 g) than leaves (0.43 g/100 g), whereas raffinose and trehalose are minor but significant oligosaccharides. The whole plant contains the highest amount of total sugar (7.2 g/100 g), while the stem has the lowest amount (5.6 g/100 g), which is in line with its lower levels of fat and protein. It is rich in organic acids, which are responsible for its antioxidant and digestive properties. The leaves (2.8 g/100 g) possessed the largest content of citric acid, which plays the greatest active role in the leaves’ extracts. Oxalic acid (maximum 1.18 g/100 g), malic acid, and ascorbic acid also contribute to the antioxidant capacity and acid taste of yerba mate. The amount of total organic acids is 10.1 g/100 g in the whole plant, indicating that it is a major component for phyto-health benefits. Yerba mate also contains minerals, fatty acids, proteins, carbohydrates, soluble sugars, and organic acids; however, their contribution to the reported pharmacological effects at customary intake levels is less directly established than that of caffeoylquinic acids, methylxanthines, flavonoids, and saponins [11,23,24,25].

Additionally, genetic and environmental factors can also affect the chemical profile of yerba mate. Investigations on various genotypes, including BRS and EC38, have indicated that phytochemical composition may differ greatly. For instance, BRS possesses higher content of caffeine and theobromine, whereas EC38 has higher levels of total flavonoids and more pronounced antioxidant activity. These results emphasize the need for appropriate genotype selection and production systems to obtain high-quality yerba mate for therapeutic or nutraceutical use [26,27].

**Table 1:** Principal bioactive constituents of *Ilex paraguariensis* relevant to the interpretation of pharmacological and nutritional studies. Reported concentrations refer to dry material or extracts and should not be interpreted as equivalent to exposure from a customary beverage [15,16,20,28,29,30,31,32].

Constituent	Class	Reported Concentration	Interpretive Relevance
Caffeine	Methylxanthine	8.7–17.5 mg/g dry weight	Major stimulant and principal dose-related safety determinant; extraction and serving practices strongly affect exposure.
Theobromine	Methylxanthine	0.5–1.5 mg/g dry weight	May contribute mild stimulant and vasoactive effects but generally occurs below caffeine.
Chlorogenic acid	Caffeoylquinic acid	28.2–44.5 mg/g dry weight	Principal candidate linking composition with antioxidant, inflammatory, and metabolic outcomes.
Neochlorogenic acid	Caffeoylquinic acid	6.1–13.2 mg/g dry weight	Contributes to the phenolic profile; relevance depends on extraction and bioavailability.
3,4-*O*-dicaffeoylquinic acid	Caffeoylquinic acid	10–17 mg/g dry weight	Potential contributor to cellular signaling and antioxidant effects; human exposure is incompletely characterized.
Rutin	Flavonol glycoside	7.1–7.8 mg/g dry weight	Supports antioxidant and vascular hypotheses, but its clinical contribution remains uncertain.
Quercetin	Flavonol	Approximately 0.5 mg/g dry weight	Mechanistically active in experimental systems, although its comparatively low abundance limits direct attribution.
Caffeic acid	Phenolic acid	0.5–1.7 mg/g dry weight	May contribute to antioxidant and inflammatory effects as a free compound and metabolite.
Mate saponins	Triterpenoid saponins	Not consistently quantified	Proposed contributors to inflammatory and lipid-related effects; standardization is limited.

## 5. Extraction and Formulation as Determinants of Biological Activity

Extraction is considered here as a determinant of pharmacological interpretation rather than as an independent catalog of technologies. Solvent polarity, temperature, pressure, extraction time, and solid-to-liquid ratio alter the relative recovery of chlorogenic acids, methylxanthines, flavonoids, saponins, and lipophilic constituents. Consequently, biological findings cannot be reliably compared without reporting extract composition and processing conditions. The following methods are, therefore, evaluated according to their relevance to yield, selectivity, food-grade applicability, and standardization (Table 2).

**Figure 2:**
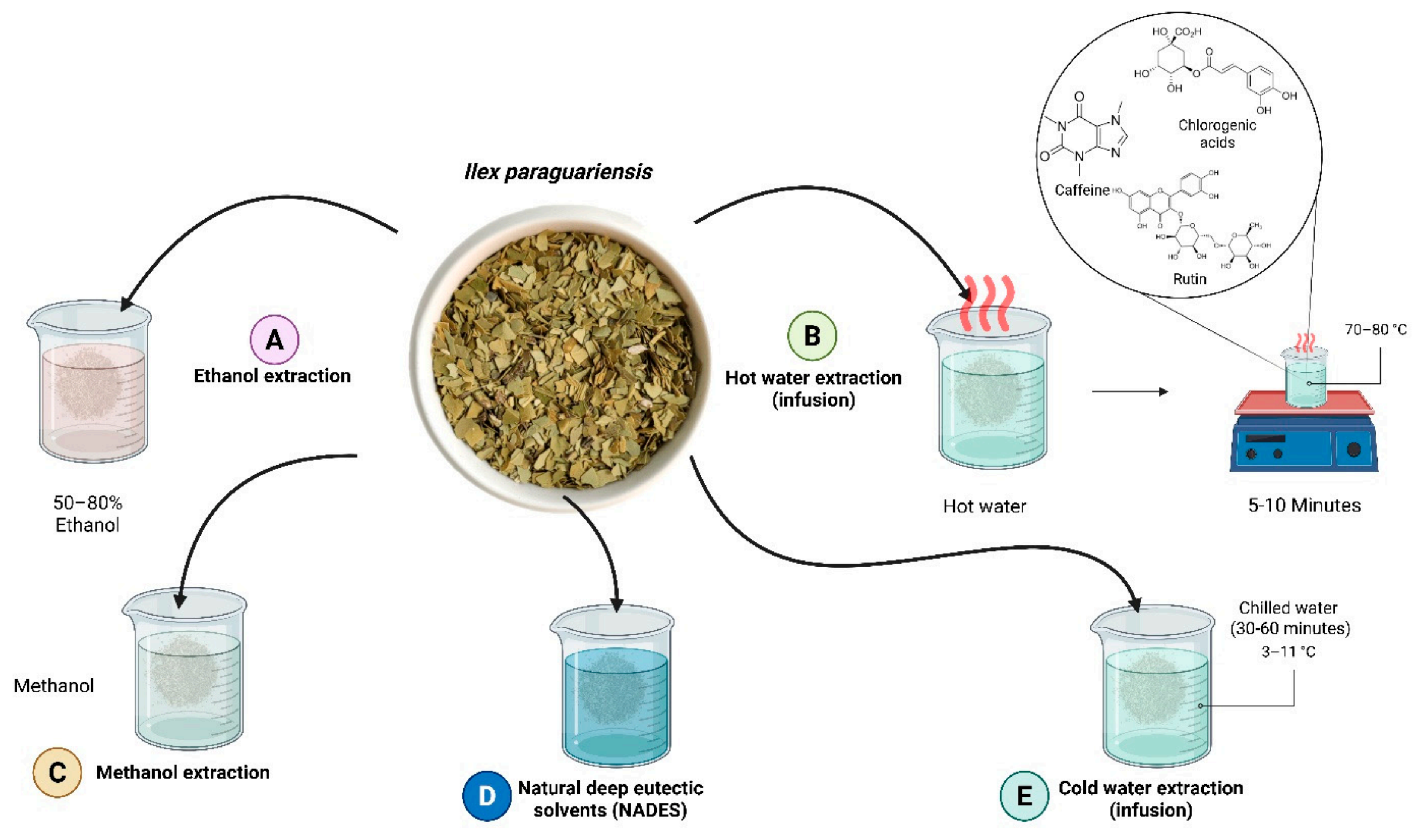
Conventional and alternative solvent extraction of caffeine from the leaves of *Ilex paraguariensis*. These compounds include ethanol extraction (**A**), hot water extraction (**B**), methanol extraction (**C**), natural deep eutectic solvents (**D**), and cold water extraction (**E**). The most important bioactive compounds (caffeine, chlorogenic acids, and rutin) are emphasized. Created in BioRender. Awlqadr, F. (2025) https://BioRender.com/k8zg715.

**Figure 3:**
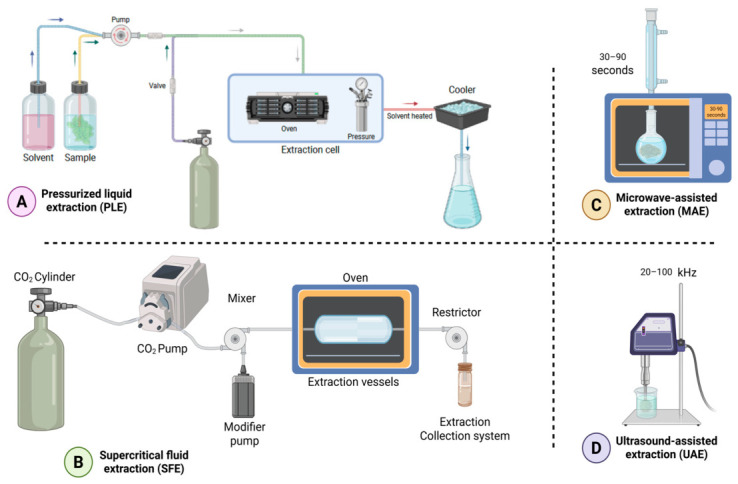
Innovative extraction technologies for *Ilex paraguariensis*: pressurized liquid extraction (PLE, (**A**)), supercritical fluid extraction (SFE, (**B**)), microwave-assisted extraction (MAE, (**C**)), and ultrasound-assisted extraction (UAE, (**D**)). These are methods that support yield, selectivity, and bioactivity retention. Created in BioRender. Awlqadr, F. (2025) https://BioRender.com/d2dkouu.

Extraction of bioactive compounds from yerba mate can be achieved through a range of conventional and advanced techniques. Each method varies in efficiency, selectivity, environmental impact, and suitability for different compounds. Various extraction methods have been employed to obtain bioactive compounds from *Ilex paraguariensis*, ranging from conventional approaches using ethanol, methanol, or water infusions to eco-friendly alternatives, such as natural deep eutectic solvents (NADES; Figure 2). In addition, advanced technologies like pressurized liquid extraction (PLE), supercritical fluid extraction (SFE), microwave-assisted extraction (MAE), and ultrasound-assisted extraction (UAE) offer efficient and sustainable strategies to maximize yield and bioactivity of key compounds (Figure 3). Recent evidence indicates that the functional value of yerba mate extracts depends not only on extraction yield but also on industrial processing, phytochemical standardization, and the gastrointestinal stability of the recovered constituents. Simulated gastrointestinal digestion studies suggest that major compounds, including chlorogenic acids and caffeine, may retain measurable stability and bioaccessibility during digestion. Nevertheless, differences in plant genotype, processing conditions, consumption format, solvent composition, and extraction parameters complicate direct comparison among studies. Consequently, standardized extraction and digestion protocols are required before compositional findings can be reliably translated into substantiated nutritional or health claims [33].

### 5.1. Traditional Aqueous Extraction

#### 5.1.1. Hot Water Infusion

Hot water extraction is employed in either the traditional method of brewing tea or the newest methods of heating water to exact temperatures for specific lengths of time to extract teas perfectly. Infusion by hot water is the oldest method for serving yerba mate, being the most common, and it serves for chimarrão and tereré. In this practice, water heated to 70–80 °C is poured over dried and crushed leaves and stems of *Ilex paraguariensis*, and the infusion time is usually 5–10 min. This method can remove the main bioactive compounds, such as caffeine, chlorogenic acids, rutin, and other flavonoids, that play a role in antioxidant, stimulant, and metabolic action of yerba mate. However, a long duration of high-temperature exposure would result in the partial degradation of thermolabile components, which might decrease the total antioxidant activity of the tested infusion. Despite this drawback, hot water extraction still appears to be a reliable choice on account of its simplicity, availability, and correspondence with traditional consumption habits from South America [34].

#### 5.1.2. Cold Water Extraction

Cold water extraction is widely used to prepare tereré, a traditional South American beverage similar to yerba mate that is consumed with chilled water. The “dunk method” involves the use of water at temperatures of 3–11 °C, for durations from 30 to 60 min. Unlike hot infusions, which is less efficient against heat-labile compounds, extraction with cold water preserves heat-sensitive flavonoids like kaempferol and catechins. Its general performance of extraction of polyphenols and antioxidants is lower than that of hot water extraction, probably because of the lower solubility and slower rate of diffusion at low temperature. However, cold beverages have always been very popular for their cool handling properties, whereas the active effects, which could be destroyed by high-heat conditions, are known for not being lost in cold extracts. This approach mirrors regional cultural practices and provides a potential complementary phytochemical profile for nutraceutical purposes [34,35].

### 5.2. Ethanol Extraction

Ethanol is one of the most used solvents in phytochemical studies of bioactive compounds present in *Ilex paraguariensis*. It does so by ‘solubilizing’ various compounds, most notably polyphenols, flavonoids, and methylxanthines, such as caffeine and theobromine. Ethanol is capable of dissolving higher bioactive concentrations than water, particularly when absorbing into hydroalcoholic systems (commonly 50–80% ethanol) where both polar and moderately non-polar compounds can be recovered. This process is also applicable for the enrichment of fractions of antioxidants, and is an extensively applied technique used to manufacture functional food ingredients, dietary supplements, and standardized herbal extracts. In addition, ethanol is a nontoxic solvent for food and drug uses and, therefore, it is more preferred in the products intended as ingested. Ethanol extracts of yerba mate have had the highest antioxidant and radical scavenging activity, which can be correlated to its increased level of chlorogenic acids and total phenolics found in several studies [35,36].

### 5.3. Methanol Extraction

Methanol is largely used in analytical phytochemistry to extract and quantify polyphenolics of antioxidative nature in *Ilex paraguariensis*. The high polarity of methanol renders it especially capable of solubilizing a variety of phenolic acids and flavonoids; therefore, methanol extracts from the same material often exhibit the highest antioxidant potential in comparative studies. This has been well-supported by the results obtained in in vitro assays, such as DPPH and ABTS, in which methanol extracts from yerba mate demonstrate a potent free radical inhibition owing to their high total phenolic content. Despite its hydrophobic nature, methanol, used for organic extracts, is toxic and is not suitable for formulations used for human treatments. It is still being used as a research and development material for the exploration, particularly for profiling phenolic fingerprints, antioxidative potential, and quality control of raw plant material [35,36].

### 5.4. Ultrasound-Assisted Extraction (UAE)

UAE is a green and rapid technique that speeds up the liquid extraction by the effect of the acoustic cavitation, where plant cell walls are disrupted. One optimized method began with the preparation of a natural eutectic solvent as a THEDES (therapeutic deep eutectic solvent) consisting of citric acid, glycerol, and water combined in a predetermined molar ratio. The dried yerba mate was subsequently blended with the THEDES at a solid:liquid ratio of 1:10 (*w*/*v*). The mixture was then subjected to sonication in an ultrasonic bath (25–40 kHz) for 30 min at 50 °C, followed by filtration, and stored at 4 °C for subsequent analyses [37]. The system, the method, and the composition improved the water solubility of the polyphenols and increased the cytotoxicity of the extract against cancer cells compared to the traditional solvents. Further, the UAE is amenable to optimization with parameters, such as temperature, solvent composition, power of ultrasound, and extraction period; thus, a versatile technique that can be applied to both small- and large-scale preparations. Its mild processing conditions also render it compatible with thermolabile compounds and thus its application in functional food ingredients and nutraceuticals [35,36].

### 5.5. Microwave-Assisted Extraction (MAE)

MAE is a technique performed by acting with microwave radiation to accelerate the heating of plant tissues and solvents, so as to improve mass transfer and to enhance intracellular compounds’ extraction. MAE has been a very interesting technique used in *Ilex paraguariensis* because it is highly efficient in the extraction of bioactive compounds, including chlorophylls, polyphenols (mainly chlorogenic acids), and other thermally stable antioxidants. One of the benefits of this approach is the high efficiency—you can reach the same extraction yields you would by hot water infusion, but orders of magnitude faster—generally timescales of 30 to 90 s. The microwave irradiation reduces the cell wall structures and hence enhances the penetration of the solvent and desorption of compounds without extended heat treatment, as in conventional methods. MAE is designed to maintain the antioxidant properties of the extract, reducing the consumption of solvent and thermal degradation and, therefore, it is a promising technique for both analytical and industrial purposes [25,35].

### 5.6. Supercritical Fluid Extraction (SFE)

SFE uses supercritical CO_2_, commonly with ethanol as a co-solvent, to recover selected constituents from *I. paraguariensis*. At certain temperature and pressure values (above 31 °C, 74 bar), CO_2_ presents some characteristics of both gas and liquid behavior, exhibiting enhanced diffusivity and solvent capacity into plant matrices. Some active constituents and moderately polar compounds, e.g., caffeine, theobromine, and some lipophilic antioxidants, are best extracted through SFE, so it is well-suited for the selective extraction of methylxanthines and essential oils. In contrast to the solvent extraction techniques, SFE has the benefit of generating solvent-free extracts since the CO_2_ evaporates completely following de-pressurization and does not leave any toxic solvent residues. That makes it particularly appealing for use in the food, pharmaceutical, or cosmetic sectors. Furthermore, SFE is a green technology, decreases solvent waste, and is tunable for improved selectivity and yield in terms of pressure, temperature, and co-solvent concentration. It has been demonstrated that SFE extracts from yerba mate conserve their high purity and stability, especially those with a higher content of caffeine [35,38].

### 5.7. Pressurized Liquid Extraction (PLE)

PLE accelerates the generation process by heating and pressurizing. Firstly, it is dried and ground into mate powder and filled in stainless-steel extraction cells. The solvent is distilled water at 10 MPa and 120 °C. The solution is retained for 20–30 min. The material is removed and, on release of pressure, is filtered. It results in a polyphenol extract that is both suitable for food and nutraceutical applications and can be used for transforming waste into value-added products in a sustainable manner. The approach offers a significant reduction of both the time required for extraction and the amount of solvent used when compared with traditional maceration or Soxhlet techniques. It also enables simultaneous extraction, purification, and quantification of compounds, such as chlorogenic acid, rutin, and caffeine, with good accuracy and precision when coupled to SPE and inline detection systems, such as HPLC-DAD and LC-MS/MS. The high-throughput capacity of PLE renders it instrumental for either analytical profiling or industrial-scale extraction of yerba mate when standardized and quality-controlled extracts are required [35,39].

### 5.8. Natural Deep Eutectic Solvents (NADES)

NADES have been recently proposed as emerging green solvents for the extraction of bioactive molecules from food or medicinal plants like *Ilex paraguariensis*. NADES are mixtures of natural, biodegradable compounds, such as organic acids and simple (mono- or di-saccharides) sugars or amino acids or polyols that can be mixed together and molten at melting temperatures well below those of the respective pure components. These solvents are polarity-adjustable for the selective solubilization of diverse phytochemicals, especially phenolic compounds. Specifically, hydrophilic NADES are efficient for extracting polar antioxidants, such as chlorogenic acid, caffeic acid, and rutin. Because NADES are nontoxic and eco-friendly, they are very appealing for the preparation of food-grade and pharmaceutical extracts from the source. Although investigations using NADES in the preparation of yerba mate extracts are relatively new, initial research showed that NADES can help obtain similar or higher amounts of polyphenols compared to traditional organic solvents, while preserving their antioxidant potential [40]. In addition, NADES avoid VOC-based procedures, which lowers health- and environmental-related risks and complies with the principles of green extraction [41,42]. Figure 2A–E illustrate *Ilex paraguariensis* leaves’ caffeine extraction from conventional and alternative solvent extraction methods. Figure 3A–D, however, represent innovative extraction technologies for *Ilex paraguariensis*.

## 6. Critical Evaluation of Pharmacological and Nutritional Evidence

The strength and translational relevance of evidence differ substantially among the biological effects attributed to *Ilex paraguariensis*. Chemical antioxidant assays, cell experiments, animal models, observational studies, and controlled human interventions represent different levels of evidence and should not be interpreted equivalently.

In the following sections, preclinical evidence refers to chemical, in vitro, in silico, invertebrate, and animal studies that investigate mechanisms or biological activity. Human evidence refers to observational studies and clinical interventions involving participants. Human biomarker changes are distinguished from demonstrated improvements in disease incidence, symptoms, progression, or other clinically meaningful outcomes.

**Figure 4:**
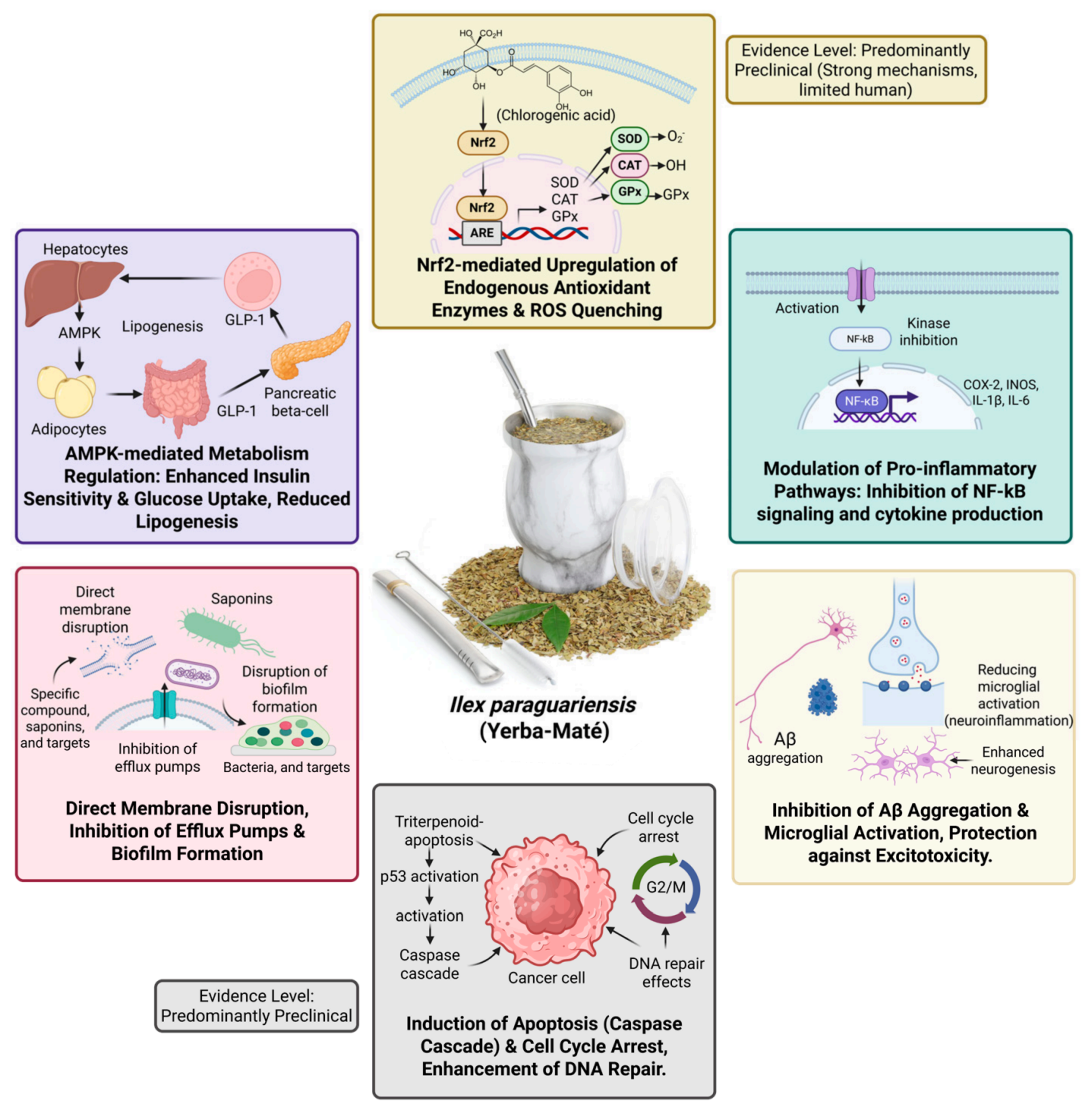
Reported biological activities of *Ilex paraguariensis* (yerba mate) according to the current evidence base. Antioxidant, anti-inflammatory, antimicrobial, metabolic, neuroprotective, and antimutagenic/anticancer effects have been described predominantly in in vitro and animal studies. Evidence from controlled human trials remains limited. Created in BioRender. Awlqadr, F. (2026) https://BioRender.com/wmz2izr.

Antioxidant, inflammatory, and metabolic mechanisms have comparatively greater biological coherence, but controlled human evidence remains limited. Antimicrobial, neuroprotective, and anticancer findings are predominantly preclinical. Accordingly, these activities are presented as areas requiring further investigation rather than as established preventive or therapeutic applications. Food product and formulation studies demonstrate technological feasibility but are not considered evidence of disease prevention or clinical efficacy [43,44,45,46,47]. The multifaceted health features associated with *Ilex paraguariensis* are attributed to its diverse pharmacological activities, including antioxidant, anti-inflammatory, antibacterial, antidiabetic, and neuroprotective properties (Figure 4).

### 6.1. Antioxidant Activity

*Ilex paraguariensis* contains chlorogenic acids, caffeic acid, quercetin, rutin, saponins, caffeine, and theobromine, all of which can contribute to antioxidant activity. In chemical and cell-based assays, yerba mate preparations scavenge reactive species, inhibit lipid peroxidation, and influence endogenous antioxidant enzymes, such as superoxide dismutase, catalase, and glutathione peroxidase [48]. DPPH, ABTS, FRAP, and ORAC assays consistently demonstrate antioxidant capacity, but these assays do not directly establish change in a biomarker, unless a true clinical outcome was measured. A limited number of human studies have reported short-term increases in plasma antioxidant capacity or decreases in malondialdehyde after consumption [49,50]; however, small sample sizes, heterogeneous preparations, short follow-up periods, and limited control of dietary confounders restrict interpretation. Evidence that yerba mate prevents or treats cardiovascular, neurodegenerative, metabolic, or malignant disease is, therefore, insufficient. Proposed links with LDL oxidation, endothelial function, insulin resistance, neuronal oxidative injury, and DNA protection remain mechanistically plausible but are supported mainly by preclinical evidence [1,51,52,53]. Extraction method, genotype, cultivation conditions, drying, roasting, dose, and beverage preparation also alter the phytochemical profile and limit comparison among studies [35,54]. Thus, antioxidant activity supports further investigation and possible use as a functional food attribute, but it should not be presented as established efficacy against chronic disease.

### 6.2. Antimicrobial Properties

The antimicrobial activity of *Ilex paraguariensis* has been evaluated mainly through in vitro experiments using aqueous, ethanolic, or other solvent extracts. Growth inhibition has been reported against several Gram-positive and Gram-negative organisms, including *Staphylococcus aureus*, *Listeria monocytogenes*, *Salmonella Enteritidis*, and *Escherichia coli*, with activity varying according to extract composition, concentration, pH, strain, and test method [55,56,57,58]. Some studies have also reported effects on biofilm formation. These findings identify yerba mate as a possible source of antimicrobial molecules and as a candidate food-preservation ingredient—they do not demonstrate efficacy for prevention or treatment of human infection. The relatively high extract concentrations used in many experiments, lack of standardized preparations, and limited information on bioavailability and toxicity further constrain clinical translation. Table 3, therefore, summarizes antimicrobial susceptibility data as preclinical evidence rather than therapeutic evidence.

Studies involving resistant bacteria, including carbapenemase-producing Gram-negative isolates, have reported bacteriostatic or bactericidal effects of ethanolic extracts and occasional in vitro interactions with antibiotics [59]. Phenolic-rich extracts derived from processing residues and extracts produced using supercritical fluids have also shown antimicrobial activity in laboratory models [44,60]. Nevertheless, these studies do not establish clinical safety, effective human dosing, pharmacokinetics, or superiority to approved antimicrobial agents. Their principal relevance is to compound discovery, food preservation, and formulation research. Claims regarding the management of antimicrobial resistance should, therefore, remain hypothesis-generating until validated in appropriate animal infection models and controlled human studies.

Nanotechnology-based formulations, including silver nanoparticles synthesized using yerba mate extracts, have also shown antimicrobial effects in vitro [61]. Because the observed activity may arise from the nanoparticles, residual silver ions, the plant-derived capping agents, or their combination, these results cannot be attributed solely to yerba mate. Safety, exposure, migration, and regulatory considerations must be resolved before food or biomedical applications can be inferred.

**Table 3:** Critical summary of preclinical antimicrobial studies of *Ilex paraguariensis*.

Organism and Experimental Model	Yerba Mate Preparation	Assay and Exposure	Main Outcome	Principal Limitations	Evidence Level	Reference
*S. aureus*, *E. coli*, *L. monocytogenes*, and *S. Enteritidis*; in vitro susceptibility testing	Extract type and chemical characterization should be verified from the original source	MIC/MBC testing; exposure duration not reported in the current manuscript	MIC values of 0.78–1.56 mg/mL; MBC of 0.78 mg/mL reported for selected organisms	Extract composition and exposure time incompletely reported; no infection model; concentrations may not be achievable through normal consumption	Low-to-moderate in vitro evidence	[56]
*E. coli* O157:H7 in broth and an apple-juice food model	Aqueous extract prepared from commercial yerba mate	MBC testing in broth and apple juice; duration should be inserted from the original study	MBC of 5–10 mg/mL in broth and 40 mg/mL in apple juice	Activity decreased substantially in the food matrix; no animal or human infection evidence	Moderate food model evidence; no clinical evidence	[58]
Carbapenem-resistant *K. pneumoniae*, *P. rettgeri*, and *P. aeruginosa* isolates	Ethanolic yerba mate extract; composition should be reported	In vitro MIC testing; duration not reported	MIC range of 1–32 mg/mL; selected interactions with antibiotics were reported	High and variable concentrations; resistant isolates were tested only in vitro; extract standardization was limited	Preliminary in vitro evidence	[59]
*S. aureus* and *E. coli*	Silver nanoparticles synthesized using yerba mate extract	In vitro inhibition assay	Antimicrobial activity was reported for the nanoparticle formulation	Activity cannot be attributed specifically to yerba mate because silver nanoparticles and residual silver ions may be responsible	Formulation-specific in vitro evidence	[61]
*Propionibacterium acnes*/*Cutibacterium acnes*; in vitro model	Methanolic extract	MIC testing; concentration 1 mg/mL	Growth inhibition and selected inflammatory effects were reported	Methanol extract is not directly food or clinically applicable; no topical or clinical validation	Preliminary in vitro evidence	

### 6.3. Anti-Inflammatory Activity

*Ilex paraguariensis* (yerba mate) is a plant with centuries of traditional use in South America and growing consumption worldwide, as a daily beverage and medicinally because of its reported or potential effects on health. The widespread use of yerba mate for medicinal proposes, especially as an anti-inflammatory remedy in South American ethnobotanical medicine, has also promoted a huge number of experimental studies that evidence and explain its therapeutic application. These anti-inflammatory actions are attributed to the large diversity of bioactive compounds present in yerba mate, such as polyphenols, caffeoylquinic acids, saponins, and alkaloids, which can act alone or interact with each other to modulate the inflammatory response in all tested disease models [47,62,63,64].

**Figure 5:**
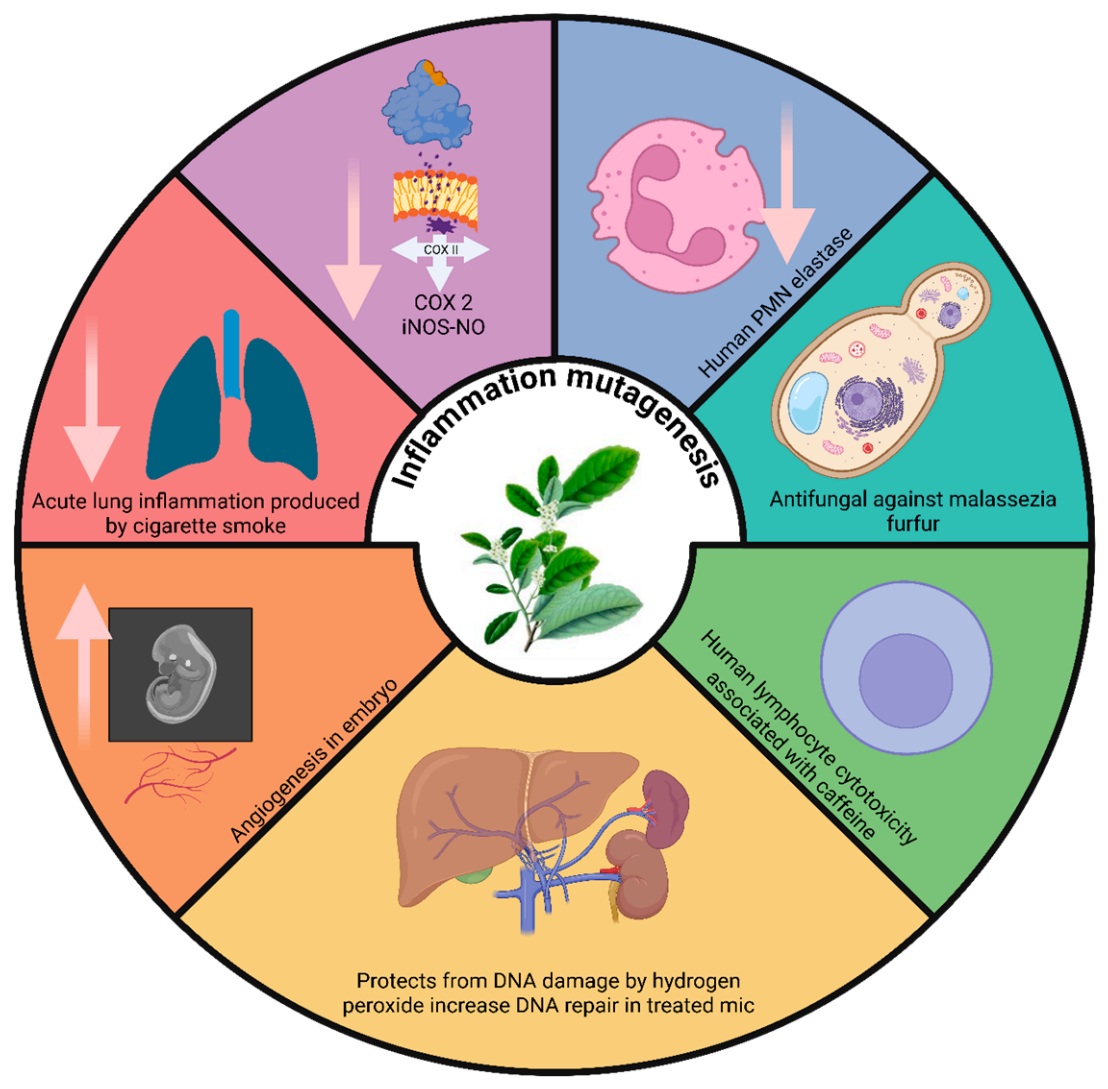
Key biological effects of *Ilex paraguariensis* (yerba mate), including anti-inflammatory, antifungal, antioxidant, cytoprotective, and pro-angiogenic activities. Created in BioRender. Awlqadr, F. (2025) https://BioRender.com/j3kz30h.

The biological activities of *Ilex paraguariensis* range from anti-inflammatory, antioxidant, antifungal, and cytoprotective, to angiogenesis modulator effects (Figure 5), which are consistent with its therapeutic potential in different systems. One of the more exciting studies recently found that yerba mate has a protective effect in inflammatory bowel disease models. Olate-Briones et al. [65] studied the action of yerba mate in a DSS-induced colitis mouse model and found that exposure to oral yerba mate reduced colitis severity and augmented the survival rate. This was associated with increased infiltration of anti-inflammatory M2 macrophages in the colon and changes in gut microbiota composition, mainly on *Lactobacillus* spp., suggesting that yerba mate promotes an immunosuppressive context, which could reduce intestinal inflammation. This anti-inflammatory action on the bowel is of particular interest due to the increasing prevalence and need for a safe, diet-based therapy in inflammatory bowel disease around the world [66].

Outside of the gastrointestinal (GI) tract, yerba mate has also shown anti-inflammatory effects in models of neurologic diseases. Herrada et al. [67] investigated the effect of yerba mate in a murine EAE model, an MS model of the disease. They found that yerba mate decreased the clinical severity, selected clinical parameters, demyelination, and infiltration of immune cells in the CNS. In addition, this treatment rescued the suppressive function of regulatory T cells (Tregs) that are important for controlling autoimmunity and inflammation, suggesting that yerba mate may have neuroprotective effects through the immune response modulation in chronic neuroinflammatory disease, such as Parkinson’s. Anti-inflammatory effects of yerba mate have also been reported in metabolic disorders like obesity and type 2 diabetes, in which chronic low-grade inflammation has emerged as a key pathogenic mechanism. Yerba mate has been found to decrease inflammatory mediators, increase insulin sensitivity, and modulate adipogenesis pathways in experimental models of obesity, suggesting its application as a dietary strategy to modulate metabolic disease [68]. This result is in agreement with the results of Alkhatib et al. [69], who highlighted the role of yerba mate and other functional foods in anti-inflammatory effects and maintaining glycemic control as part of preventative and management strategies against diabetes.

The anti-inflammatory effects of yerba mate have been partially attributed to its high concentration in caffeoylquinic acids and other phenolic compounds. Puangpraphant et al. [70] reported that 3,4-*O*-dicaffeoylquinic acid derivatives (from the leaves of yerba mate) inhibited LPS-induced macrophage inflammation by inhibiting the nuclear translocation of NF-κB subunits, which is an essential transcription factor for inflammatory requirements. Furthermore, these compounds also triggered apoptosis in colon cancer cells, suggesting a connection between their anti-inflammatory and putative antineoplastic activities. Supporting the systemic anti-inflammatory effects of yerba mate, Correa et al. [71] investigated its impact in rats with adjuvant-induced arthritis, used as a model for chronic inflammation. Treatment attenuated the hepatic and cerebral ROS production, lipoperoxidation, and protein damage, and restored the antioxidant enzymes, indicating that yerba mate has both anti-inflammatory and antioxidant effects, which could protect against systemic inflammatory diseases. In a paired in vitro study, Farias et al. [48] showed that phenolic-rich yerba mate aqueous infusion extracts strongly inhibited NO and TNF-α production in activated macrophages, and important bioactive compounds, such as theobromine, were even more potent than those of anti-inflammatory drugs, such as the standard drug, dexamethasone. Interestingly, the anti-inflammatory property of yerba mate also applies to its topical uses. Tsai et al. [64] have reported on the growth inhibitory effects and pro-inflammatory cytokine reduction by yerba mate extract against Propionibacterium acnes, which suggests that it could be integrated as a natural anti-inflammatory agent in the therapeutic management of acne. These local and systemic actions have been ascribed to the particular capacity of yerba mate phenolics to interact with cell membranes and modulate inflammatory signaling, as the molecular dynamics analysis of phenolics-lipid bilayer interactions has shown [72].

Moreover, yerba mate’s anti-inflammatory properties might be related to the inhibition of the formation of advanced glycation end products (AGEs) that can cause inflammation and tissue damage, particularly in metabolic disorders. It was previously shown by Bains and Gugliucci [73] that yerba mate extract strongly reduced the formation of AGEs in vitro in digestion-like assays, which explains its systemic anti-inflammatory effects. Of critical relevance is the manner in which yerba mate is prepared and its anti-inflammatory effect. Mesquita et al. [74] compared chimarrão, tereré, and yerba mate tea, among other traditional preparations, and demonstrated that chimarrão had higher phenolic content and better antioxidant and anti-inflammatory effects in animal models. Similarly, Khaksar et al. [75] stressed that the brewing process plays a pivotal role in the extraction of bioactive compounds, and the main ones responsible for the anti-inflammatory effects of yerba mate were caffeoylquinic acids. The anti-inflammatory effects of yerba mate have been recorded in human studies as well. Petrilli et al. [76] demonstrated that phenolics from yerba mate increased inflammatory and oxidative biomarkers in people with HIV/AIDS, who are presented with systemic inflammation. Moreover, Cittadini et al. [77] showed that phenolic compounds from yerba mate reached the brains of lung adenocarcinoma mice, decreased the levels of IL-6, and supplied neuroprotection, thus supporting the systemic bioavailability and anti-inflammatory activity of yerba mate.

New findings indicate that yerba mate modulates certain relevant inflammatory pathways in neurodegenerative diseases. Santos et al. [78] reported that yerba mate extract suppressed the activation of NLRP3 inflammasome in microglia and macrophages, attenuated the oxidative damage, and protected neuronal cells through neuroprotective anti-inflammatory effects. Altogether, the overall evidence from animal, cellular, and limited human studies is consistent with the anti-inflammatory effects of yerba mate on different systems, including the GI tract, the CNS, metabolic tissues, and skin. The high content of phytochemicals, such as caffeoylquinic acids, saponins, and polyphenols, form the basis for its immunomodulatory and anti-inflammatory properties. Overall, the available evidence supports anti-inflammatory mechanisms in cellular and animal models, but controlled human evidence remains insufficient to recommend yerba mate for the prevention or treatment of inflammatory diseases. Table 4 summarizes these studies in terms of study design, objective, and effective doses, demonstrating the diversity of research from which its immunomodulatory action is supported.

### 6.4. Anti-Obesity, Lipid-Lowering, and Antidiabetic Effects

Yerba mate (*Ilex paraguariensis*) has gained considerable scientific attention for its anti-obesity, lipid-lowering, and antidiabetic properties. The beneficial health effects of yerba mate are primarily attributed to its rich content of bioactive compounds, including polyphenols, saponins, xanthines, such as caffeine and theobromine, and various vitamins and minerals, which act synergistically to modulate metabolic processes. Substantial preclinical and clinical research has demonstrated the potential of yerba mate in reducing body weight and improving lipid profiles. Gambero and Ribeiro [68] provided an extensive review highlighting how yerba mate suppresses adipocyte differentiation, reduces triglyceride accumulation, and modulates key signaling pathways involved in adipogenesis and insulin resistance. The authors concluded that yerba mate not only helps regulate lipid metabolism but also exerts anti-inflammatory effects that are critical in obesity management. Furthermore, yerba mate has been shown to modulate gene expression patterns that are typically dysregulated in obesity, restoring them to more favorable profiles associated with improved metabolic health. In addition to its effects on adiposity, yerba mate exhibits promising lipid-lowering properties. Masson et al. [79] conducted a systematic review and meta-analysis to evaluate the association between yerba mate consumption and blood lipid levels. Although results from observational and experimental studies were somewhat conflicting, quantitative analysis of 7 studies involving 378 participants revealed trends toward reduced total cholesterol, LDL cholesterol, and triglyceride levels. However, the authors noted that the existing studies were small and sometimes inconclusive, highlighting the need for larger, well-controlled trials to confirm these effects. The antidiabetic properties of yerba mate have also been extensively described. In the study of Derosa et al. [80], which included individuals with deglycation, a nutraceutical formulation of yerba mate, white mulberry, and chromium picolinate was able to promote a significant improvement in glycemic control. Subjects receiving the supplement were found to have significantly lower fasting plasma glucose, postprandial glucose, and glycated hemoglobin levels, and higher insulin sensitivity than those on placebo. The supplement also had a positive effect on lipids, such as total cholesterol, LDL-cholesterol, and triglycerides, further supporting the role of yerba mate in metabolic regulation.

The mechanisms responsible for these metabolic improvements have been investigated in animal models. Jang et al. [81] showed that the hepatocellular lipid accumulation and oxidative stress in rats under carbon tetrachloride-induced liver injury was markedly attenuated after treatment with 70% ethanol extract of yerba mate. Hepatoprotective and lipid-lowering actions were ascribed to bioactive compounds, such as chlorogenic acid and cynarine, that regulate key enzymes for lipid metabolism and antioxidant pathways. Recent findings suggest that yerba mate also influences gut hormones related to glucose homeostasis. Cooper-Leavitt et al. [21] examined the effect of yerba mate on the incretin in mice and reported that consumption of yerba mate significantly promoted expression of glucagon-like peptide-1 (GLP-1), an insulin stimulator and glycemic efficiency enhancer. This effect was partly attributable to gut-microbiota-mediated metabolism of ferulic acid, a major polyphenol in yerba mate, revealing a complex and dynamic tripartite interplay among dietary components, gut microbiota, and host metabolites. Besides its direct metabolic actions, yerba mate has been described as a functional food able to prevent metabolic alterations when consumed in a daily diet. Alkhatib et al. [69] emphasized the contribution of polyphenol-rich functional foods, such as yerba mate, to improved antioxidant status, inflammation, insulin sensitivity, and lipid profile, leading to antidiabetic effect or glycemic regulation. The authors also proposed integration of functional foods into personalized lifestyle interventions for optimizing metabolic health. Last but not least, the metabolic actions of yerba mate are not just demonstrated by clinical trials but also in population-based studies. Choi et al. [82] studied the dietary composition of school children in Argentina and reported that low intake of polyphenolic compounds such as yerba mate may be a contributing factor on susceptibility to obesity and chronic disease, thus suggesting a protective effect against metabolic disease on populations at risk from yerba mate. Overall, yerba mate may influence selected metabolic biomarkers, but the available evidence is insufficient to establish efficacy for the prevention or treatment of obesity, dyslipidemia, diabetes, or cardiovascular disease.

**Figure 6:**
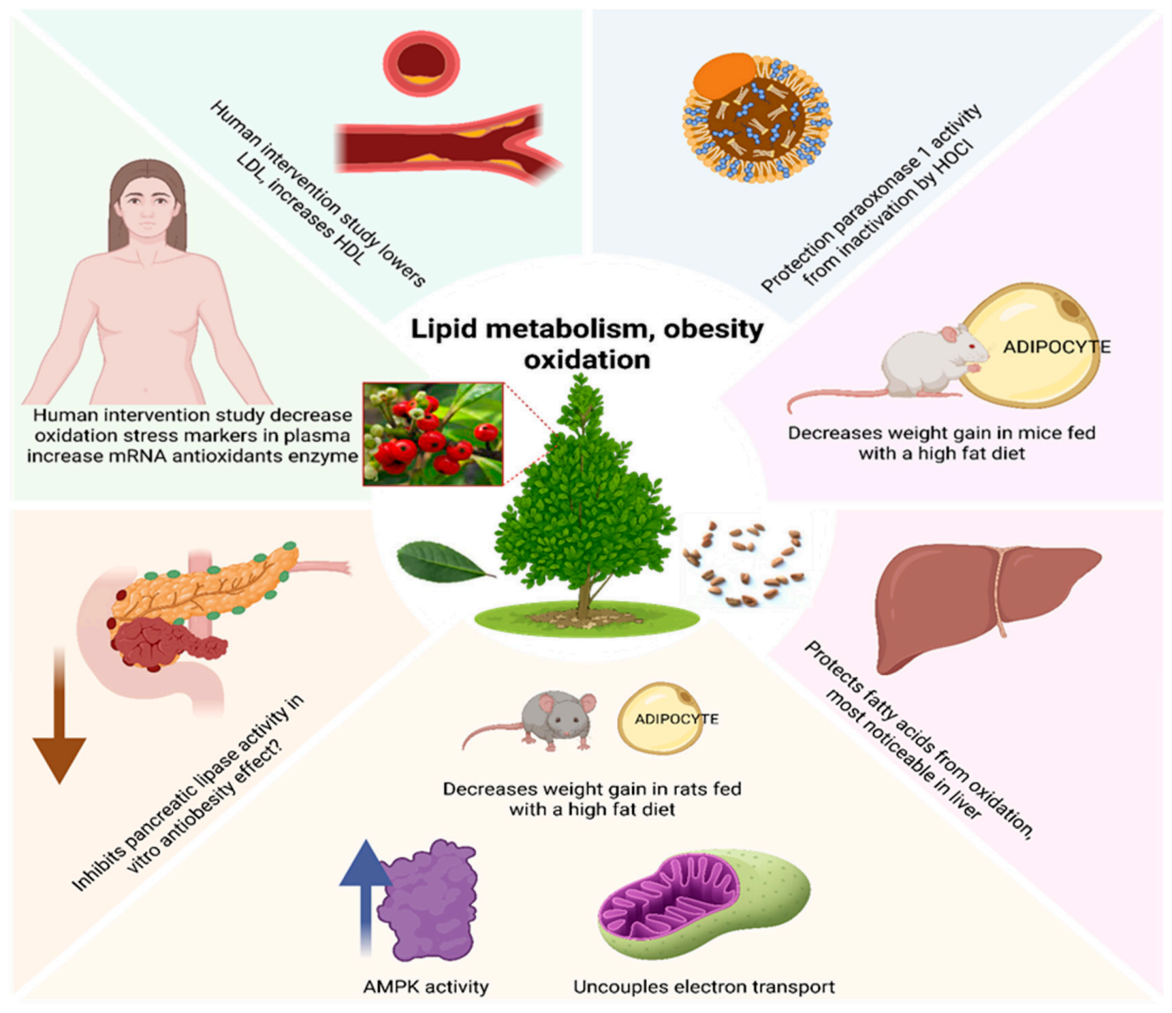
Metabolic- and oxidative-stress-related effects reported for *Ilex paraguariensis*. Most mechanistic- and body-weight-related findings derive from animal or cellular models, whereas controlled human evidence remains limited and does not establish treatment or prevention of metabolic disease. Created in BioRender. Awlqadr, F. (2025) https://BioRender.com/6023rca.

Despite these promising findings from current studies, additional large-scale, well-designed human studies are needed to confirm these effects as well as to determine optimal formulas and doses for health benefits. A wide variety of animal models, clinical trials, and systematic reviews reported beneficial metabolic effects of *Ilex paraguariensis*. As illustrated in Table 5, these studies are in favor of its anti-obesity, lipid-lowering, and antidiabetic activity among the studies. As shown in Figure 6, *Ilex paraguariensis* has several positive effects on metabolism and on the control of oxidative stress, such as improvements in lipid profiles, weight loss in HFD models, and improvement in mitochondrial activity.

### 6.5. Neuroprotective and Cognitive Evidence of Ilex paraguariensis

Proposed neuroprotective effects of yerba mate are based largely on its methylxanthines and polyphenols and their antioxidant, inflammatory, mitochondrial, and acetylcholinesterase-related activities [30,87,88,89]. However, evidence concerning isolated methylxanthines or individual polyphenols should not automatically be attributed to yerba mate as a complete beverage or extract.

Preclinical studies have reported effects on neuronal oxidative damage, acetylcholinesterase activity, inflammatory signaling, mitochondrial function, and dopaminergic neuron survival in cell, invertebrate, and rodent models [52,90,91]. These findings support biological plausibility and may help identify mechanisms for further research. Nevertheless, they do not demonstrate that yerba mate delays cognitive decline or prevents or treats Alzheimer’s disease, Parkinson’s disease, or other neurodegenerative disorders in humans.

Evidence from controlled human studies evaluating cognition or neurodegenerative disease outcomes is currently insufficient. Acute alertness associated with caffeine consumption should also be distinguished from long-term neuroprotection. Therefore, yerba mate should presently be described as a source of compounds with preclinical neurobiological activity rather than as a clinically validated neuroprotective intervention.

### 6.6. Antimutagenic and Anticancer Evidence of Ilex paraguariensis

Antimutagenic and anticancer activities attributed to *I. paraguariensis* have been evaluated mainly using chemical assays, cultured cells, and animal models. Experimental studies have reported reduced oxidative DNA damage, modulation of mutagenicity assays, altered detoxification pathways, induction of apoptosis, and cell-cycle arrest [32,92,93,94,95]. These findings provide mechanistic and preclinical evidence only. The use of human-derived colon, esophageal, breast, or prostate cancer cell lines does not constitute human clinical evidence. Concentrations that induce apoptosis in isolated cells may not be achievable or safe in human tissues following customary beverage consumption. No adequate clinical trials currently demonstrate that yerba mate prevents cancer, improves treatment response, reduces recurrence, or prolongs survival.

Epidemiological interpretation is complicated by beverage temperature, smoking, alcohol use, dietary practices, consumption patterns, and possible exposure to polycyclic aromatic hydrocarbons generated during some drying processes [96,97]. Therefore, associations between mate consumption and upper aerodigestive cancer cannot be attributed solely to the plant itself, but neither can risk be dismissed without considering preparation temperature and product contamination. The current evidence supports further investigation of antimutagenic and anticancer mechanisms but does not justify chemopreventive or therapeutic claims. Risk reduction measures should include avoidance of very hot consumption and improved control of smoke-derived contaminants. Overall, available evidence neither establishes intrinsic carcinogenicity of the plant nor demonstrates anticancer efficacy. Risk assessment should distinguish botanical composition from exposure to very-high beverage temperatures and smoke-derived contaminants. More clinical studies are required to confirm these effects in humans and to determine their safe consumption level (Table 6).

### 6.7. Uses of Ilex paraguariensis in the Food Industry

**Figure 7:**
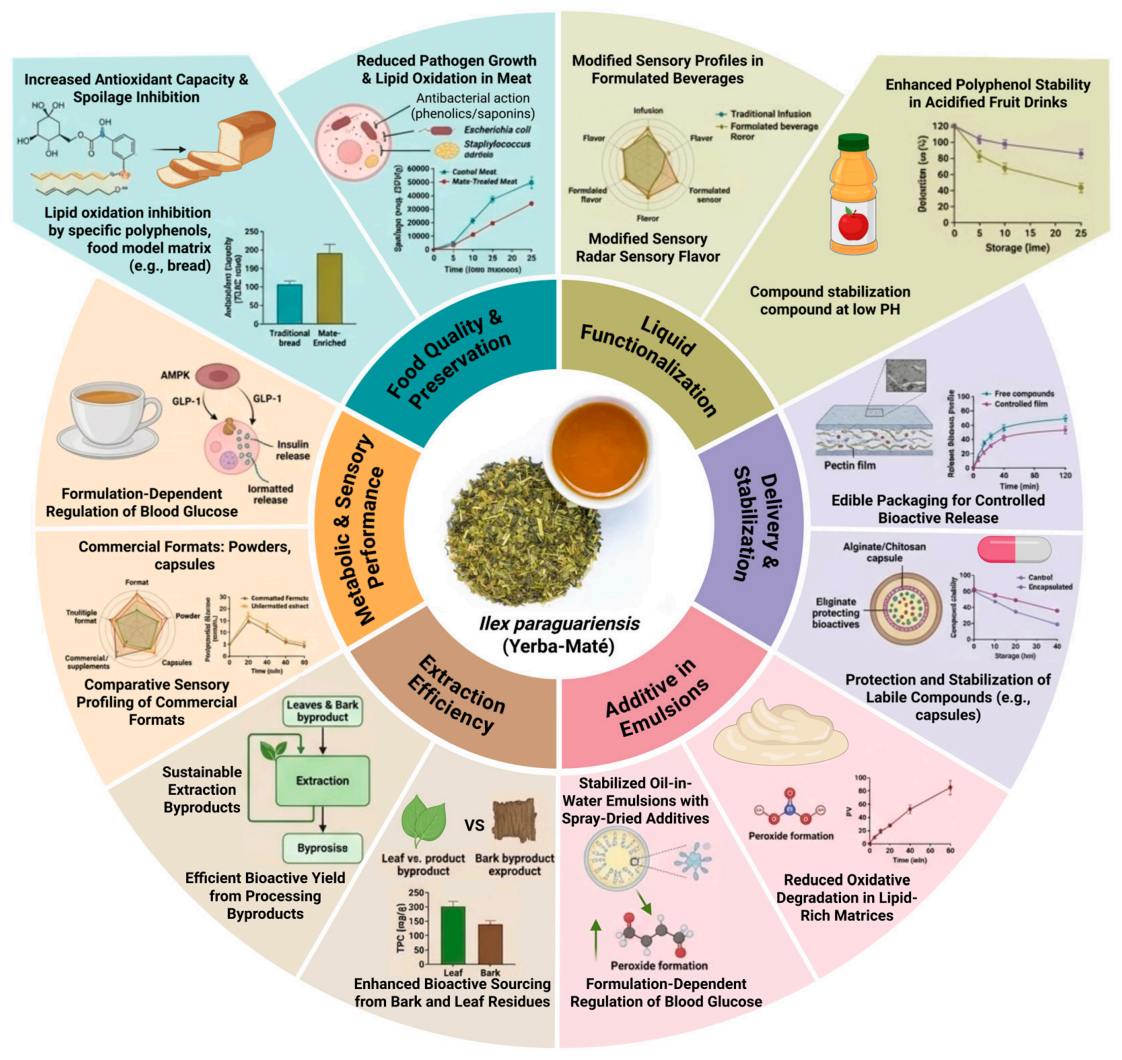
Functional applications of *Ilex paraguariensis* (yerba mate) in food systems. Created in BioRender. Awlqadr, F. (2026) https://biorender.com/1mz129i.

Yerba mate (*Ilex paraguariensis*) is increasingly investigated as a natural functional ingredient because its polyphenols, methylxanthines, and saponins may provide antioxidant, antimicrobial, and sensory benefits in food systems [11,35,98]. In meat and poultry products, yerba mate extracts have been reported to reduce lipid oxidation and microbial spoilage while maintaining acceptable sensory characteristics, suggesting potential as partial alternatives to synthetic preservatives [99,100]. Their incorporation into yogurt, bakery products, functional beverages, and acidified fruit drinks has also increased antioxidant capacity and polyphenol content, although product-specific effects on texture, flavor, microbial viability, and consumer acceptance require further evaluation [11,58,101,102]. Antimicrobial activity against foodborne microorganisms, including *Escherichia coli* O157:H7 and *Staphylococcus aureus*, further supports investigation of yerba mate as a natural preservation ingredient [98]. Yerba mate processing residues, including leaves and bark, are also rich in phenolic compounds and may be converted into antioxidant and antimicrobial food ingredients using green extraction technologies [103,104]. Spray-dried extracts and pelletized powders have demonstrated acceptable physicochemical stability and retention of polyphenols during controlled storage, supporting their incorporation into solid and powdered foods [105,106]. Although bioactive compounds may remain stable during some processing operations, extraction, formulation, storage, and food-matrix interactions can substantially influence their final functionality [107,108]. Proposed metabolic benefits, including effects on oxidative stress, cholesterol, and glucose regulation, remain formulation-dependent and should not be interpreted as established clinical effects of yerba-mate-enriched foods [109,110]. Commercial formats include teas, functional beverages, concentrates, powders, capsules, and supplements. Their food applications, product formats, and reported antioxidant, antimicrobial, sensory, and metabolic outcomes are summarized and illustrated conceptually in Figure 7.

**Table 6:** Applications of *Ilex paraguariensis* (yerba mate) in functional foods, packaging, and antimicrobial formulations.

Type of Yerba Mate Used	Formulation	Aim of Study	Reference
Aqueous extract (75 g and 150 g/500 mL)	Bread	Functional enrichment of bread with yerba mate extract	[111]
Instant powder formulation	Infusion simulation for sensory profiling	Flavor analysis and sensory evaluation of yerba-mate-like products	[112]
*Ilex paraguariensis* extract	Supplement in high-fat diet	Prevent weight gain and improve metabolic health	[110]
Aqueous extracts from commercial tea	Antimicrobial screening in food pathogens	Use as a natural food antimicrobial	[58]
Residue from harvest pruning (bark/leaves)	Bioactive compound profiling	Utilization of yerba mate waste in industrial applications	[111]
Aqueous extract	pH-modified apple juice	Pathogen inhibition in apple juice model	[99]
Spray-dried extract	Pellets (extrusion/spheronization)	Development of stable yerba mate pellets	[105]
Packaging	Pectin films with *Ilex paraguariensis* extract and sorbitol	Improve UV protection and water barrier, add bioactives, use general food wrapping	[45]
Enrichment	Alginate/chitosan capsules with yerba mate extract	Polyphenol delivery and antioxidant enhancement used in instant soup	[113]
Additive	Spray-dried yerba mate extract with maltodextrin	Enhance oxidative stability used for mayonnaise	[114]
Packaging	Chitosan/PVA/Starch film with Ilex extract	Antimicrobial activity against *S. aureus* in beef packaging	[115]
Packaging	Cornstarch film with yerba mate fruit extract	Improve antioxidant activity and biodegradability; biodegradable packaging for perishables	[116]

## 7. Safety, Toxicological Considerations, and Regulatory Perspectives

Although *Ilex paraguariensis* is widely consumed as a traditional beverage and is generally well-tolerated when used moderately, its safety must be evaluated in relation to dose, preparation conditions, processing contaminants, individual susceptibility, and concurrent medication use. Caffeine is the principal constituent of safety relevance, accompanied by lower amounts of theobromine and, in some preparations, theophylline; however, actual exposure varies considerably according to cultivar, leaf-to-stem ratio, processing method, serving size, water temperature, infusion duration, and the repeated addition of water during traditional consumption. The European Food Safety Authority concluded that single caffeine doses of up to 200 mg and total habitual intakes of up to 400 mg/day generally do not raise safety concerns for healthy non-pregnant adults, whereas pregnant and lactating women should limit total caffeine intake from all dietary sources to 200 mg/day [117]. Accordingly, the safety of yerba mate cannot be judged solely by the number of servings because consumers may simultaneously obtain caffeine from coffee, tea, energy drinks, chocolate, supplements, and medications. Excessive exposure may cause insomnia, nervousness, anxiety, tremor, headache, gastrointestinal discomfort, palpitations, transient increases in blood pressure, and tachycardia, particularly among caffeine-naïve or caffeine-sensitive individuals and those with anxiety disorders, sleep disturbances, uncontrolled hypertension, or cardiac arrhythmias [118,119]. Pregnancy requires particular caution because caffeine crosses the placenta and is eliminated more slowly during gestation, while fetal caffeine metabolism is limited. Although the available evidence is predominantly observational and remains vulnerable to residual confounding and reverse causation, professional and regulatory guidance generally recommends maintaining total maternal intake below 200 mg/day [117,120]. A separate and clinically important risk concerns beverage temperature. The International Agency for Research on Cancer classified the consumption of very hot beverages, defined as beverages consumed at temperatures above approximately 65 °C, as probably carcinogenic to humans (Group 2A), based mainly on evidence relating repeated thermal injury of the esophageal mucosa to an increased risk of esophageal squamous cell carcinoma [121,122]. This classification refers principally to the temperature of the beverage rather than establishing that the botanical constituents of yerba mate are inherently carcinogenic; nevertheless, traditional mate is often consumed repeatedly at high temperatures, making cooling before consumption a practical risk reduction measure. Processing-related contamination also deserves consideration because direct smoke-drying can deposit polycyclic aromatic hydrocarbons (PAHs), including benzo[a]pyrene and other potentially carcinogenic compounds, on leaves and stems. Analytical studies have demonstrated substantial variability in PAH concentrations among brands, production batches, and processing procedures, indicating that exposure is strongly influenced by manufacturing practice rather than being uniform across all yerba mate products [123,124]. Therefore, indirect heating, smoke-free drying, validated supplier controls, and routine monitoring of PAH markers should be prioritized in commercial production. Potential herb–drug interactions remain insufficiently characterized specifically for yerba mate, but its caffeine content provides a biologically plausible basis for interactions involving hepatic CYP1A2 metabolism and additive central nervous system or cardiovascular stimulation. Particular caution may be appropriate in individuals taking caffeine-containing medicines, stimulants, monoamine oxidase inhibitors, theophylline, clozapine, tizanidine, or potent CYP1A2 inhibitors, although the clinical relevance depends on dose, treatment duration, genetic differences in caffeine metabolism, smoking status, and comorbidities [118,119]. Available intervention studies involving yerba mate are generally short and often include small samples and chemically heterogeneous preparations; consequently, an absence of reported serious adverse events should not be interpreted as confirmation of long-term safety, especially for concentrated extracts or supplements that may provide exposures different from customary beverages [36,125]. From a regulatory perspective, yerba mate products should be evaluated as complex caffeinated botanical preparations, with attention to botanical authentication, good agricultural and manufacturing practices, microbial and chemical contaminants, pesticide residues, PAHs, batch-to-batch caffeine variability, accurate serving information, and warnings for vulnerable populations. Overall, moderate consumption of quality-controlled yerba mate at temperatures below the very-hot range is likely to be tolerated by most healthy adults, but definitive universal safe-dose recommendations cannot be made independently of preparation method and cumulative caffeine intake. Pregnant or lactating women, children, individuals with caffeine sensitivity or relevant cardiovascular and psychiatric conditions, and patients taking potentially interacting medicines should, therefore, apply additional caution, while future studies should use standardized products, quantify caffeine and contaminants, report adverse events systematically, and evaluate longer-term clinically relevant safety outcomes [36,117].

## 8. Conclusions and Future Perspectives

The most defensible interpretation of the current evidence is that *I. paraguariensis* is a bioactive caffeinated beverage and a potential source of standardized functional ingredients, not an established pharmacological therapy. The strongest mechanistic support concerns oxidative stress and inflammatory pathways, while the most clinically relevant—but still preliminary—human findings concern lipid, glycemic, and body-weight-related outcomes. Antimicrobial, neuroprotective, and anticancer effects remain predominantly preclinical. Across all domains, variation in plant material, extraction, chemical composition, dose, and formulation limits comparability and may explain part of the inconsistency among studies. Safety conclusions must remain preparation-specific because caffeine exposure, serving pattern, consumption temperature, smoke-derived PAHs, medication use, and individual susceptibility differ considerably among consumers and products. Future research should, therefore, prioritize chemically characterized preparations, exposure-relevant doses, adequately powered and sufficiently long controlled trials, clinically meaningful endpoints, and systematic safety monitoring. This narrower evidence-based framework distinguishes plausible nutritional value from broad therapeutic claims and provides a clearer basis for responsible functional food and nutraceutical development.

## Figures and Tables

**Table 2:** Comparative overview of conventional and emerging extraction methods for bioactive compounds, including operating conditions, advantages, limitations, scalability, and food/pharmaceutical applicability.

Extraction Method	Solvent/System	Typical Conditions	Principal Target Compounds	Advantages	Disadvantages	Scalability	Food/Pharmaceutical Applicability
Hot water infusion	Water	70–80 °C; 5–10 min	Caffeine, chlorogenic acids, rutin, water-soluble phenolics	Simple, inexpensive, food-grade, consistent with traditional consumption	Limited selectivity; possible degradation of thermolabile compounds; relatively dilute extract	High	High for beverages and food products
Cold water extraction	Water	3–11 °C; 30–60 min	Caffeine, selected phenolics, heat-sensitive constituents	Preserves thermolabile compounds; food-grade	Slower mass transfer and generally lower extraction efficiency	High	High for beverages; limited for concentrated extracts
Hydroethanolic extraction	50–80% ethanol–water	Ambient to moderate temperature; minutes to hours	Phenolic acids, flavonoids, methylxanthines	Broad polarity range; relatively high recovery; ethanol is food-compatible	Solvent recovery required; flammability; residual-solvent control	High	High after solvent removal and validation
Methanolic extraction	Methanol or methanol–water	Ambient temperature; variable duration	Phenolic acids and flavonoids for analytical profiling	High analytical extraction efficiency	Toxic; unsuitable for food or direct pharmaceutical use	Low for products; high for laboratory analysis	Analytical use only
Ultrasound-assisted extraction	Water, ethanol–water, or NADES	25–40 kHz; approximately 30 min; ≤50 °C	Chlorogenic acids, flavonoids, caffeine	Short extraction time; reduced solvent consumption; suitable for thermolabile compounds	Scale-up depends on reactor geometry; possible local heating and compound degradation at excessive power	Moderate to high	High when food-grade solvents are used
Microwave-assisted extraction	Water or polar organic solvents	Usually seconds to minutes; controlled microwave power	Polyphenols, caffeine, chlorophyll-associated compounds	Rapid heating, high mass-transfer rate, low solvent use	Risk of overheating; less suitable for highly thermolabile constituents; equipment cost	Moderate to high	High with validated food-grade systems
Pressurized liquid extraction	Water or ethanol under pressure	Approximately 10 MPa; up to 120 °C; 20–30 min	Chlorogenic acids, rutin, caffeine	Rapid, reproducible, reduced solvent volume, compatible with inline analysis	High equipment cost; elevated temperature may alter sensitive compounds	Moderate	High for standardized food and nutraceutical extracts
Supercritical fluid extraction	Supercritical CO_2_, often with ethanol co-solvent	>31 °C and >74 bar; pressure-dependent	Caffeine, theobromine, lipophilic compounds	Solvent-free final extract; tunable selectivity; low oxygen exposure	High capital cost; limited recovery of highly polar compounds without co-solvent	High in specialized facilities	High for pharmaceutical and premium food ingredients
Natural deep eutectic solvent extraction	Organic acid/sugar/polyol/amino-acid-based NADES	Composition-dependent; often combined with stirring or ultrasound	Chlorogenic acid, caffeic acid, rutin, other polar phenolics	Low volatility, tunable polarity, potentially biodegradable and food-compatible	High viscosity; difficult solvent removal; incomplete toxicological and regulatory characterization for some systems	Emerging/moderate	Promising, but formulation-specific safety validation is required

**Table 4:** Critical summary of preclinical and limited human evidence concerning the anti-inflammatory effects of *Ilex paraguariensis*.

Study Design and Model	Preparation	Dose and Duration	Main Outcomes	Principal Limitations	Reference
DSS-induced colitis in mice	Orally administered yerba mate preparation; chemical characterization should be stated	Dose and duration not reported in the current manuscript	Reduced selected disease severity measures; increased M2 macrophage polarization; altered gut microbiota composition	Animal model; unspecified dose and composition; no human inflammatory bowel disease evidence	[64]
Experimental autoimmune encephalomyelitis in mice	Oral yerba mate preparation	Dose and duration should be obtained from the original source	Reduced disease severity, inflammatory cell infiltration, and demyelination; modulation of regulatory T cells	Disease-specific animal model; no controlled human neurological evidence	[66]
Adjuvant-induced arthritis in rats	Aqueous yerba mate extract	400 and 800 mg/kg/day; duration should be inserted	Reduced inflammatory and oxidative markers and restored selected antioxidant enzyme activities	High animal doses; no human confirmation; translation to customary intake is uncertain	[70]
LPS-stimulated macrophages; in vitro	Isolated 3,4-*O*-dicaffeoylquinic acid from yerba mate	Concentration range and exposure duration should be reported	Inhibited NF-κB nuclear translocation and selected inflammatory mediators	Isolated compound and cellular evidence; concentrations may not reflect dietary exposure	[69]
Human intervention involving individuals with HIV/AIDS	Phenolic intervention involving yerba mate and chocolate; exact formulation should be clarified	Sample size, dose, and duration should be inserted	Changes in selected inflammatory and oxidative biomarkers were reported	Combined intervention; small and specialized population; biomarker findings do not establish clinical benefit	[75]
Microglial/macrophage cellular models with complementary in vivo experiments	Yerba mate extract	15 µg/mL in vitro; in vivo dose and duration should be inserted	Suppressed NLRP3 inflammasome activation, reduced oxidative damage, and protected neuronal cells	Mixed experimental models; no human evidence; extract characterization and dose relevance require clarification	[76]

**Table 5:** Critical summary of clinical and preclinical evidence on the metabolic effects of *Ilex paraguariensis* (yerba mate).

Level of Evidence	Principal Limitations	Main Outcomes	Yerba Mate Preparation	Study Design and Model	Reference
Moderate for lipid biomarkers; insufficient for clinical cardiovascular benefit	Small number of studies and participants; heterogeneous products, doses, populations, and study designs; possible publication bias	Pooled analyses suggested possible reductions in total cholesterol, LDL cholesterol, and triglycerides; however, findings were inconsistent across individual studies	Yerba mate beverages or preparations differing across the included studies	Systematic review and meta-analysis of seven human studies; 378 participants	[78]
Low for yerba-mate-specific efficacy	Effects cannot be attributed specifically to yerba mate because a multi-ingredient formulation was used; limited generalizability to traditional mate consumption	Improvements were reported in selected glycemic and metabolic variables	Multi-ingredient supplement containing an *I. paraguariensis* leaf extract standardized to 2% 1-deoxynojirimycin, white mulberry, and chromium picolinate	Randomized controlled trial in non-diabetic adults with dysglycemia	[79]
Moderate preclinical evidence	Animal model; dose equivalence to habitual human consumption is uncertain; no clinical endpoints	Reduced body-weight gain, white adipose tissue mass, adipocyte size, plasma triglycerides, total cholesterol, free fatty acids, insulin resistance, and hepatic lipid accumulation; increased energy expenditure and thermogenic gene expression	Dietary yerba mate supplementation	In vivo study in C57BL/6J mice with high-fat-diet-induced obesity	[81]
Moderate preclinical evidence	Preclinical model; limited characterization of extract composition; findings cannot be directly extrapolated to humans	Reduced adipocyte differentiation and lipid accumulation, body-weight gain, serum cholesterol, triglycerides, and glucose; metabolic changes were associated with altered food intake and energy expenditure	Yerba mate extract	In vivo study in C57BL/6J mice fed a high-fat diet	[83]
Moderate preclinical evidence for lipid lowering	Animal study; lack of human confirmation; extract standardization and bioavailability may differ from brewed mate	Reduced serum lipid variables and improved selected markers of diet-induced dyslipidemia	Standardized *I. paraguariensis* extracts	In vivo study in rats receiving a high-fat diet	[84]
Low-to-moderate preclinical evidence	Specialized animal model; unclear relevance to males, premenopausal women, or habitual consumers; no clinical outcomes	Altered white adipose tissue morphology and metabolic characteristics	Yerba mate beverage or extract	In vivo study in ovariectomized rats, used as a model of estrogen-deficiency-associated metabolic changes	[85]
Moderate mechanistic preclinical evidence	Short-duration animal study; ex vivo concentrations may not reflect human tissue exposure; clinical relevance remains unconfirmed	Improved glucose tolerance and glucose-stimulated insulin secretion; increased IRS-1 and PI3K expression; decreased oxidative stress and inflammatory gene expression in pancreatic islets	Aqueous yerba mate infusion; dry extract and isolated phenolics used in islet experiments	In vivo dietary-intervention study in rats, with complementary ex vivo experiments in isolated pancreatic islets	[86]
Contextual evidence only; not direct efficacy evidence	Narrative design; heterogeneous evidence; no pooled effect estimate; conclusions largely based on preclinical studies	Summarized possible effects on adipogenesis, triglyceride accumulation, inflammation, insulin signaling, and obesity-related gene expression	Multiple yerba mate preparations	Narrative review of cellular, animal, and limited human evidence	[67]

## Data Availability

No new data were created or analyzed in this study. Data sharing is not applicable to this article.

## Supplementary Materials

The following supporting information can be downloaded at: https://www.mdpi.com/article/10.3390/nu18152535/s1, Supplementary Material S1: *Ilex paraguariensis* (Yerba Mate): A Narrative Review of Bioactive Compounds, Biological Activities, and Green Extraction Technologies.

## Abbreviations

The following abbreviations are used in this manuscript:

ABTS	2,2′-Azino-bis(3-ethylbenzothiazoline-6-sulfonic acid)
AChE	Acetylcholinesterase
AD	Alzheimer’s disease
AGEs	Advanced glycation end products
AMPK	AMP-activated protein kinase
As(III)	Trivalent inorganic arsenic (arsenite)
As(V)	Pentavalent inorganic arsenic (arsenate)
BMI	Body mass index
CNS	Central nervous system
COX-2	Cyclooxygenase-2
CYP1A2	Cytochrome P450 1A2
DMA	Dimethylarsinic acid
DNA	Deoxyribonucleic acid
DPPH	2,2-Diphenyl-1-picrylhydrazyl
DSS	Dextran sulfate sodium
EAE	Experimental autoimmune encephalomyelitis
FRAP	Ferric-reducing antioxidant power
GI	Gastrointestinal
GLP-1	Glucagon-like peptide-1
HDL	High-density lipoprotein
HFD	High-fat diet
HIV/AIDS	Human immunodeficiency virus/acquired immunodeficiency syndrome
HOCl	Hypochlorous acid
HPLC–DAD	High-performance liquid chromatography with diode-array detection
IL-6	Interleukin-6
iNOS	Inducible nitric oxide synthase
IRS-1	Insulin receptor substrate 1
kHz	Kilohertz
KPC	*Klebsiella pneumoniae* carbapenemase
LC–MS/MS	Liquid chromatography–tandem mass spectrometry
LDL	Low-density lipoprotein
LPS	Lipopolysaccharide
M2	Alternatively activated M2 macrophage phenotype
MAE	Microwave-assisted extraction
MBC	Minimum bactericidal concentration
MBL	Metallo-β-lactamase
MEDLINE	Medical Literature Analysis and Retrieval System Online
MIC	Minimum inhibitory concentration
MPa	Megapascal
mRNA	Messenger ribonucleic acid
MS	Multiple sclerosis
NADES	Natural deep eutectic solvents
NCDs	Non-communicable diseases
NDM	New Delhi metallo-β-lactamase
NF-κB	Nuclear factor kappa B
NLRP3	NOD-, LRR-, and pyrin-domain-containing protein 3
NO	Nitric oxide
ORAC	Oxygen radical absorbance capacity
PAHs	Polycyclic aromatic hydrocarbons
PD	Parkinson’s disease
PI3K	Phosphoinositide 3-kinase
PLE	Pressurized liquid extraction
PMN	Polymorphonuclear neutrophil
PUFA	Polyunsaturated fatty acid
PVA	Poly(vinyl alcohol)
ROS	Reactive oxygen species
SFE	Supercritical fluid extraction
SPE	Solid-phase extraction
THEDES	Therapeutic deep eutectic solvent
TNF-α	Tumor necrosis factor alpha
Treg(s)	Regulatory T cell(s)
UAE	Ultrasound-assisted extraction
UPLC–QTOF–MS	Ultra-performance liquid chromatography–quadrupole time-of-flight mass spectrometry
UV	Ultraviolet
VIM	Verona integron-encoded metallo-β-lactamase
VOC(s)	Volatile organic compound(s)
*w*/*v*	Weight per volume
