# Supplementary material for: Ilex paraguariensis (Yerba Mate): A Narrative Review of Bioactive Compounds, Biological Activities, and Green Extraction Technologies
Source: Nutrients. 2026 Aug 4;18(15):2535. doi: 10.3390/nu18152535 (PMC13468038; doi:10.3390/nu18152535)
Supplement: Supplementary file 1 [file nutrients-18-02535-s001.zip › nutrients-4450262-supplementary.pdf]

# Supplementary Material S1

## *Ilex paraguariensis* (Yerba Mate): A Narrative Review of Bioactive Compounds, Biological Activities, and Green Extraction Technologies

Databases: PubMed/MEDLINE, Scopus, and Web of Science Core Collection

**Purpose and use.** This appendix provides database-specific, reproducible search syntax. The searches are structured around one species block and five thematic blocks. For transparent reporting, each species–theme combination should be executed separately, exported in full, merged in EndNote and Zotero, and deduplicated using DOI, PMID, title, author, year, and journal metadata.

## Table S1. Search log to be completed after execution

| Database/platform | Coverage searched | Final search date | Records retrieved | Export format |
|---|---|---|---|---|
| **PubMed/MEDLINE** | Title/Abstract + MeSH | [DD Month 2025] | 262 | RIS |
| **Scopus** | TITLE-ABS-KEY | [DD Month 2025] | 2,051 | RIS/CSV |
| **Web of Science Core Collection** | Topic (title, abstract, author keywords, Keywords Plus) | [DD Month 2025] | 96 | RIS |

## A. PubMed/MEDLINE

Platform: PubMed. The species search combines the specific MeSH heading with title/abstract synonyms. Thematic concepts are searched in title and abstract fields. Run the five species–theme combinations separately in PubMed Advanced Search History.

**S1. Species block**

```
("Ilex paraguariensis"[Mesh] OR "Ilex paraguariensis"[tiab] OR "I. paraguariensis"[tiab]
OR "yerba mate"[tiab] OR "yerba maté"[tiab] OR "erva mate"[tiab] OR "erva-mate"[tiab] OR
"Paraguay tea"[tiab] OR "mate tea"[tiab] OR "maté tea"[tiab] OR chimarrao[tiab] OR
chimarrão[tiab] OR terere[tiab] OR tereré[tiab])
```

**S2. Composition block**

```
(phytochem*[tiab] OR "chemical composition"[tiab] OR "chemical profile"[tiab] OR "chemical
characterization"[tiab] OR compositional[tiab] OR polyphenol*[tiab] OR phenolic*[tiab] OR
"chlorogenic acid"[tiab] OR "chlorogenic acids"[tiab] OR "caffeoylquinic acid"[tiab] OR
"caffeoylquinic acids"[tiab] OR flavonoid*[tiab] OR methylxanthine*[tiab] OR
caffeine[tiab] OR theobromine[tiab] OR saponin*[tiab] OR mineral*[tiab] OR nutrient*[tiab]
OR "proximate composition"[tiab] OR "fatty acid"[tiab] OR "fatty acids"[tiab] OR "organic
acid"[tiab] OR "organic acids"[tiab] OR sugar*[tiab] OR metabolom*[tiab] OR
volatilom*[tiab])
```

**S3. Biological and health effects block**

```
(pharmacolog*[tiab] OR bioactiv*[tiab] OR antioxidant*[tiab] OR "oxidative stress"[tiab]
OR anti-inflammatory[tiab] OR inflammation[tiab] OR inflammatory[tiab] OR
antimicrobial*[tiab] OR antibacter*[tiab] OR antifung*[tiab] OR antiviral*[tiab] OR
antidiabet*[tiab] OR glycemic[tiab] OR glycaemic[tiab] OR glucose[tiab] OR insulin*[tiab]
OR hypolipid*[tiab] OR lipid*[tiab] OR cholesterol[tiab] OR anti-obesity[tiab] OR
antiobesity[tiab] OR obes*[tiab] OR "body weight"[tiab] OR adipos*[tiab] OR
cardiovascular[tiab] OR cardioprotect*[tiab] OR neuroprotect*[tiab] OR cognit*[tiab] OR
anticancer[tiab] OR antitumor[tiab] OR antimutagen*[tiab] OR cytotox*[tiab] OR
clinical[tiab] OR trial[tiab] OR human*[tiab] OR bioavailab*[tiab] OR bioaccessib*[tiab]
OR pharmacokinetic*[tiab] OR microbiot*[tiab] OR "glucagon-like peptide 1"[tiab] OR "GLP-
1"[tiab])
```

**S4. Safety and toxicity block**

```
(safety[tiab] OR toxic*[tiab] OR "adverse effect"[tiab] OR "adverse effects"[tiab] OR
contaminant*[tiab] OR "polycyclic aromatic hydrocarbon"[tiab] OR "polycyclic aromatic
hydrocarbons"[tiab] OR PAH[tiab] OR PAHs[tiab] OR carcinogen*[tiab] OR "cancer risk"[tiab]
OR "very hot"[tiab] OR "beverage temperature"[tiab] OR "caffeine exposure"[tiab] OR
interaction*[tiab] OR pregnancy[tiab] OR lactation[tiab] OR "heavy metal"[tiab] OR "heavy
metals"[tiab] OR pesticide*[tiab] OR mycotoxin*[tiab])
```

**S5. Processing and extraction block**

```
(extract*[tiab] OR infusion*[tiab] OR decoction*[tiab] OR brew*[tiab] OR solvent*[tiab] OR
"green extraction"[tiab] OR "ultrasound-assisted extraction"[tiab] OR "ultrasound assisted
extraction"[tiab] OR UAE[tiab] OR "microwave-assisted extraction"[tiab] OR "microwave
assisted extraction"[tiab] OR MAE[tiab] OR "supercritical fluid extraction"[tiab] OR
SFE[tiab] OR "pressurized liquid extraction"[tiab] OR PLE[tiab] OR "accelerated solvent
extraction"[tiab] OR ASE[tiab] OR "natural deep eutectic solvent"[tiab] OR "natural deep
eutectic solvents"[tiab] OR NADES[tiab] OR "deep eutectic solvent"[tiab] OR "deep eutectic
solvents"[tiab] OR maceration[tiab] OR Soxhlet[tiab] OR drying[tiab] OR roasting[tiab] OR
"smoke drying"[tiab] OR "smoke-drying"[tiab] OR blanching[tiab] OR sapeco[tiab] OR
storage[tiab] OR encapsulat*[tiab] OR formulat*[tiab] OR "spray drying"[tiab] OR
lyophiliz*[tiab])
```

**S6. Botanical factors and applications block**

```
(leaf[tiab] OR leaves[tiab] OR stem*[tiab] OR root*[tiab] OR fruit*[tiab] OR seed*[tiab]
OR residue*[tiab] OR byproduct*[tiab] OR "by-product"[tiab] OR "by-products"[tiab] OR
morpholog*[tiab] OR anatom*[tiab] OR genotype*[tiab] OR cultivar*[tiab] OR
cultivation[tiab] OR agronom*[tiab] OR "functional food"[tiab] OR "functional foods"[tiab]
OR nutraceutical*[tiab] OR standardi*[tiab] OR "quality control"[tiab] OR "food
application"[tiab] OR "food applications"[tiab] OR "food product"[tiab] OR "food
products"[tiab] OR preservative*[tiab] OR sensory[tiab] OR stability[tiab] OR "shelf
life"[tiab] OR fermentation[tiab] OR kombucha[tiab])
```

**S7. Species combined with composition**

```
#1 AND #2
```

**S8. Species combined with biological and health effects**

```
#1 AND #3
```

**S9. Species combined with safety and toxicity**

```
#1 AND #4
```

**S10. Species combined with processing and extraction**

```
#1 AND #5
```

**S11. Species combined with botanical factors and applications**

```
#1 AND #6
```

**S12. Union of all thematic searches**

```
#7 OR #8 OR #9 OR #10 OR #11
```

**S13. Final date- and language-limited set**

```
#12 AND ("2015/01/01"[Date - Publication] : "2025/06/30"[Date - Publication]) AND
English[Language]
```

## Optional single-run PubMed master query

```
("Ilex paraguariensis"[Mesh] OR "Ilex paraguariensis"[tiab] OR "I. paraguariensis"[tiab]
OR "yerba mate"[tiab] OR "yerba maté"[tiab] OR "erva mate"[tiab] OR "erva-mate"[tiab] OR
"Paraguay tea"[tiab] OR "mate tea"[tiab] OR "maté tea"[tiab] OR chimarrao[tiab] OR
chimarrão[tiab] OR terere[tiab] OR tereré[tiab]) AND ((phytochem*[tiab] OR "chemical
composition"[tiab] OR "chemical profile"[tiab] OR "chemical characterization"[tiab] OR
compositional[tiab] OR polyphenol*[tiab] OR phenolic*[tiab] OR "chlorogenic acid"[tiab] OR
"chlorogenic acids"[tiab] OR "caffeoylquinic acid"[tiab] OR "caffeoylquinic acids"[tiab]
OR flavonoid*[tiab] OR methylxanthine*[tiab] OR caffeine[tiab] OR theobromine[tiab] OR
saponin*[tiab] OR mineral*[tiab] OR nutrient*[tiab] OR "proximate composition"[tiab] OR
"fatty acid"[tiab] OR "fatty acids"[tiab] OR "organic acid"[tiab] OR "organic acids"[tiab]
OR sugar*[tiab] OR metabolom*[tiab] OR volatilom*[tiab]) OR (pharmacolog*[tiab] OR
bioactiv*[tiab] OR antioxidant*[tiab] OR "oxidative stress"[tiab] OR anti-
inflammatory[tiab] OR inflammation[tiab] OR inflammatory[tiab] OR antimicrobial*[tiab] OR
antibacter*[tiab] OR antifung*[tiab] OR antiviral*[tiab] OR antidiabet*[tiab] OR
glycemic[tiab] OR glycaemic[tiab] OR glucose[tiab] OR insulin*[tiab] OR hypolipid*[tiab]
OR lipid*[tiab] OR cholesterol[tiab] OR anti-obesity[tiab] OR antiobesity[tiab] OR
obes*[tiab] OR "body weight"[tiab] OR adipos*[tiab] OR cardiovascular[tiab] OR
cardioprotect*[tiab] OR neuroprotect*[tiab] OR cognit*[tiab] OR anticancer[tiab] OR
antitumor[tiab] OR antimutagen*[tiab] OR cytotox*[tiab] OR clinical[tiab] OR trial[tiab]
OR human*[tiab] OR bioavailab*[tiab] OR bioaccessib*[tiab] OR pharmacokinetic*[tiab] OR
microbiot*[tiab] OR "glucagon-like peptide 1"[tiab] OR "GLP-1"[tiab]) OR (safety[tiab] OR
toxic*[tiab] OR "adverse effect"[tiab] OR "adverse effects"[tiab] OR contaminant*[tiab] OR
```

```
"polycyclic aromatic hydrocarbon"[tiab] OR "polycyclic aromatic hydrocarbons"[tiab] OR
PAH[tiab] OR PAHs[tiab] OR carcinogen*[tiab] OR "cancer risk"[tiab] OR "very hot"[tiab] OR
"beverage temperature"[tiab] OR "caffeine exposure"[tiab] OR interaction*[tiab] OR
pregnancy[tiab] OR lactation[tiab] OR "heavy metal"[tiab] OR "heavy metals"[tiab] OR
pesticide*[tiab] OR mycotoxin*[tiab]) OR (extract*[tiab] OR infusion*[tiab] OR
decoction*[tiab] OR brew*[tiab] OR solvent*[tiab] OR "green extraction"[tiab] OR
"ultrasound-assisted extraction"[tiab] OR "ultrasound assisted extraction"[tiab] OR
UAE[tiab] OR "microwave-assisted extraction"[tiab] OR "microwave assisted
extraction"[tiab] OR MAE[tiab] OR "supercritical fluid extraction"[tiab] OR SFE[tiab] OR
"pressurized liquid extraction"[tiab] OR PLE[tiab] OR "accelerated solvent
extraction"[tiab] OR ASE[tiab] OR "natural deep eutectic solvent"[tiab] OR "natural deep
eutectic solvents"[tiab] OR NADES[tiab] OR "deep eutectic solvent"[tiab] OR "deep eutectic
solvents"[tiab] OR maceration[tiab] OR Soxhlet[tiab] OR drying[tiab] OR roasting[tiab] OR
"smoke drying"[tiab] OR "smoke-drying"[tiab] OR blanching[tiab] OR sapeco[tiab] OR
storage[tiab] OR encapsulat*[tiab] OR formulat*[tiab] OR "spray drying"[tiab] OR
lyophiliz*[tiab]) OR (leaf[tiab] OR leaves[tiab] OR stem*[tiab] OR root*[tiab] OR
fruit*[tiab] OR seed*[tiab] OR residue*[tiab] OR byproduct*[tiab] OR "by-product"[tiab] OR
"by-products"[tiab] OR morpholog*[tiab] OR anatom*[tiab] OR genotype*[tiab] OR
cultivar*[tiab] OR cultivation[tiab] OR agronom*[tiab] OR "functional food"[tiab] OR
"functional foods"[tiab] OR nutraceutical*[tiab] OR standardi*[tiab] OR "quality
control"[tiab] OR "food application"[tiab] OR "food applications"[tiab] OR "food
product"[tiab] OR "food products"[tiab] OR preservative*[tiab] OR sensory[tiab] OR
stability[tiab] OR "shelf life"[tiab] OR fermentation[tiab] OR kombucha[tiab])) AND
("2015/01/01"[Date - Publication] : "2025/06/30"[Date - Publication]) AND
English[Language]
```

## B. Scopus

Platform: Scopus Advanced Search. Searches are restricted to title, abstract, and keyword fields using TITLE-ABS-KEY. Run each species–theme combination separately and apply the year and language limits shown below.

**S1. Species block**

```
TITLE-ABS-KEY ("Ilex paraguariensis" OR "I. paraguariensis" OR "yerba mate" OR "yerba
maté" OR "erva mate" OR "erva-mate" OR "Paraguay tea" OR "mate tea" OR "maté tea" OR
chimarrao OR chimarrão OR terere OR tereré)
```

**S2. Composition block**

```
TITLE-ABS-KEY (phytochem* OR "chemical composition" OR "chemical profile" OR "chemical
characterization" OR compositional OR polyphenol* OR phenolic* OR "chlorogenic acid*" OR
"caffeoylquinic acid*" OR flavonoid* OR methylxanthine* OR caffeine OR theobromine OR
saponin* OR mineral* OR nutrient* OR "proximate composition" OR "fatty acid*" OR "organic
acid*" OR sugar* OR metabolom* OR volatilom*)
```

**S3. Biological and health effects block**

```
TITLE-ABS-KEY (pharmacolog* OR bioactiv* OR antioxidant* OR "oxidative stress" OR anti-
inflammatory OR inflamm* OR antimicrobial* OR antibacter* OR antifung* OR antiviral* OR
antidiabet* OR glycemic OR glycaemic OR glucose OR insulin* OR hypolipid* OR lipid* OR
cholesterol OR anti-obesity OR antiobesity OR obes* OR "body weight" OR adipos* OR
cardiovascular OR cardioprotect* OR neuroprotect* OR cognit* OR anticancer OR antitumor OR
antimutagen* OR cytotox* OR clinical OR trial OR human* OR bioavailab* OR bioaccessib* OR
pharmacokinetic* OR microbiot* OR "glucagon-like peptide 1" OR "GLP-1")
```

**S4. Safety and toxicity block**

```
TITLE-ABS-KEY (safety OR toxic* OR "adverse effect*" OR contaminant* OR "polycyclic
aromatic hydrocarbon*" OR PAH OR PAHs OR carcinogen* OR "cancer risk" OR "very hot" OR
"beverage temperature" OR "caffeine exposure" OR interaction* OR pregnancy OR lactation OR
"heavy metal*" OR pesticide* OR mycotoxin*)
```

**S5. Processing and extraction block**

```
TITLE-ABS-KEY (extract* OR infusion* OR decoction* OR brew* OR solvent* OR "green
extraction" OR "ultrasound-assisted extraction" OR "ultrasound assisted extraction" OR UAE
OR "microwave-assisted extraction" OR "microwave assisted extraction" OR MAE OR
"supercritical fluid extraction" OR SFE OR "pressurized liquid extraction" OR PLE OR
"accelerated solvent extraction" OR ASE OR "natural deep eutectic solvent*" OR NADES OR
"deep eutectic solvent*" OR maceration OR Soxhlet OR drying OR roasting OR "smoke drying"
OR "smoke-drying" OR blanching OR sapeco OR storage OR encapsulat* OR formulat* OR "spray
drying" OR lyophiliz*)
```

**S6. Botanical factors and applications block**

```
TITLE-ABS-KEY (leaf OR leaves OR stem* OR root* OR fruit* OR seed* OR residue* OR
byproduct* OR "by-product*" OR morpholog* OR anatom* OR genotype* OR cultivar* OR
cultivation OR agronom* OR "functional food*" OR nutraceutical* OR standardi* OR "quality
control" OR "food application*" OR "food product*" OR preservative* OR sensory OR
stability OR "shelf life" OR fermentation OR kombucha)
```

**S7. Species combined with composition and limits**

```
Search History > Combine Queries: Search 1 AND Search 2; then apply PUBYEAR > 2014,
PUBYEAR < 2025, and English language.
```

**S8. Species combined with biological and health effects and limits**

```
Search History > Combine Queries: Search 1 AND Search 3; then apply PUBYEAR > 2014,
PUBYEAR < 2025, and English language.
```

**S9. Species combined with safety and toxicity and limits**

```
Search History > Combine Queries: Search 1 AND Search 4; then apply PUBYEAR > 2014,
PUBYEAR < 2025, and English language.
```

**S10. Species combined with processing and extraction and limits**

```
Search History > Combine Queries: Search 1 AND Search 5; then apply PUBYEAR > 2014,
PUBYEAR < 2025, and English language.
```

**S11. Species combined with botanical factors and applications and limits**

```
Search History > Combine Queries: Search 1 AND Search 6; then apply PUBYEAR > 2014,
PUBYEAR < 2025, and English language.
```

**S12. Union of all Scopus thematic searches**

```
#7 OR #8 OR #9 OR #10 OR #11
```

## Optional single-run Scopus master query

```
TITLE-ABS-KEY ("Ilex paraguariensis" OR "I. paraguariensis" OR "yerba mate" OR "yerba
maté" OR "erva mate" OR "erva-mate" OR "Paraguay tea" OR "mate tea" OR "maté tea" OR
chimarrao OR chimarrão OR terere OR tereré) AND TITLE-ABS-KEY ((phytochem* OR "chemical
composition" OR "chemical profile" OR "chemical characterization" OR compositional OR
polyphenol* OR phenolic* OR "chlorogenic acid*" OR "caffeoylquinic acid*" OR flavonoid* OR
methylxanthine* OR caffeine OR theobromine OR saponin* OR mineral* OR nutrient* OR
"proximate composition" OR "fatty acid*" OR "organic acid*" OR sugar* OR metabolom* OR
volatilom*) OR (pharmacolog* OR bioactiv* OR antioxidant* OR "oxidative stress" OR anti-
inflammatory OR inflamm* OR antimicrobial* OR antibacter* OR antifung* OR antiviral* OR
antidiabet* OR glycemic OR glycaemic OR glucose OR insulin* OR hypolipid* OR lipid* OR
cholesterol OR anti-obesity OR antiobesity OR obes* OR "body weight" OR adipos* OR
cardiovascular OR cardioprotect* OR neuroprotect* OR cognit* OR anticancer OR antitumor OR
antimutagen* OR cytotox* OR clinical OR trial OR human* OR bioavailab* OR bioaccessib* OR
pharmacokinetic* OR microbiot* OR "glucagon-like peptide 1" OR "GLP-1") OR (safety OR
toxic* OR "adverse effect*" OR contaminant* OR "polycyclic aromatic hydrocarbon*" OR PAH
OR PAHs OR carcinogen* OR "cancer risk" OR "very hot" OR "beverage temperature" OR
"caffeine exposure" OR interaction* OR pregnancy OR lactation OR "heavy metal*" OR
pesticide* OR mycotoxin*) OR (extract* OR infusion* OR decoction* OR brew* OR solvent* OR
"green extraction" OR "ultrasound-assisted extraction" OR "ultrasound assisted extraction"
OR UAE OR "microwave-assisted extraction" OR "microwave assisted extraction" OR MAE OR
"supercritical fluid extraction" OR SFE OR "pressurized liquid extraction" OR PLE OR
"accelerated solvent extraction" OR ASE OR "natural deep eutectic solvent*" OR NADES OR
"deep eutectic solvent*" OR maceration OR Soxhlet OR drying OR roasting OR "smoke drying"
OR "smoke-drying" OR blanching OR sapeco OR storage OR encapsulat* OR formulat* OR "spray
drying" OR lyophiliz*) OR (leaf OR leaves OR stem* OR root* OR fruit* OR seed* OR residue*
OR byproduct* OR "by-product*" OR morpholog* OR anatom* OR genotype* OR cultivar* OR
cultivation OR agronom* OR "functional food*" OR nutraceutical* OR standardi* OR "quality
control" OR "food application*" OR "food product*" OR preservative* OR sensory OR
stability OR "shelf life" OR fermentation OR kombucha)) AND PUBYEAR > 2014 AND PUBYEAR <
2025 AND (LIMIT-TO(LANGUAGE, "English"))
```

## C. Web of Science Core Collection

Platform: Web of Science Core Collection Advanced Search. Topic searches (TS=) cover title, abstract, author keywords, and Keywords Plus. Run the five species–theme combinations separately and combine them in Search History.

**S1. Species block**

```
TS=("Ilex paraguariensis" OR "I. paraguariensis" OR "yerba mate" OR "yerba maté" OR "erva mate" OR "erva-mate" OR "Paraguay tea" OR "mate tea" OR "maté tea" OR chimarrao OR chimarrão OR terere OR tereré)
```

**S2. Composition block**

```
TS=(phytochem* OR "chemical composition" OR "chemical profile" OR "chemical characterization" OR compositional OR polyphenol* OR phenolic* OR "chlorogenic acid*" OR "caffeoylquinic acid*" OR flavonoid* OR methylxanthine* OR caffeine OR theobromine OR saponin* OR mineral* OR nutrient* OR "proximate composition" OR "fatty acid*" OR "organic acid*" OR sugar* OR metabolom* OR volatilom*)
```

**S3. Biological and health effects block**

```
TS=(pharmacolog* OR bioactiv* OR antioxidant* OR "oxidative stress" OR anti-inflammatory OR inflamm* OR antimicrobial* OR antibacter* OR antifung* OR antiviral* OR antidiabet* OR glycemic OR glycaemic OR glucose OR insulin* OR hypolipid* OR lipid* OR cholesterol OR anti-obesity OR antiobesity OR obes* OR "body weight" OR adipos* OR cardiovascular OR cardioprotect* OR neuroprotect* OR cognit* OR anticancer OR antitumor OR antimutagen* OR cytotox* OR clinical OR trial OR human* OR bioavailab* OR bioaccessib* OR pharmacokinetic* OR microbiot* OR "glucagon-like peptide 1" OR "GLP-1")
```

**S4. Safety and toxicity block**

```
TS=(safety OR toxic* OR "adverse effect*" OR contaminant* OR "polycyclic aromatic hydrocarbon*" OR PAH OR PAHs OR carcinogen* OR "cancer risk" OR "very hot" OR "beverage temperature" OR "caffeine exposure" OR interaction* OR pregnancy OR lactation OR "heavy metal*" OR pesticide* OR mycotoxin*)
```

**S5. Processing and extraction block**

```
TS=(extract* OR infusion* OR decoction* OR brew* OR solvent* OR "green extraction" OR "ultrasound-assisted extraction" OR "ultrasound assisted extraction" OR UAE OR "microwave-assisted extraction" OR "microwave assisted extraction" OR MAE OR "supercritical fluid extraction" OR SFE OR "pressurized liquid extraction" OR PLE OR "accelerated solvent extraction" OR ASE OR "natural deep eutectic solvent*" OR NADES OR "deep eutectic solvent*" OR maceration OR Soxhlet OR drying OR roasting OR "smoke drying" OR "smoke-drying" OR blanching OR sapeco OR storage OR encapsulat* OR formulat* OR "spray drying" OR lyophiliz*)
```

**S6. Botanical factors and applications block**

```
TS=(leaf OR leaves OR stem* OR root* OR fruit* OR seed* OR residue* OR byproduct* OR "by-product*" OR morpholog* OR anatom* OR genotype* OR cultivar* OR cultivation OR agronom* OR "functional food*" OR nutraceutical* OR standardi* OR "quality control" OR "food application*" OR "food product*" OR preservative* OR sensory OR stability OR "shelf life" OR fermentation OR kombucha)
```

**S7. Species combined with composition and limits**

```
#1 AND #2 AND PY=2015-2025 AND LA=(English)
```

**S8. Species combined with biological and health effects and limits**

```
#1 AND #3 AND PY=2015-2025 AND LA=(English)
```

**S9. Species combined with safety and toxicity and limits**

```
#1 AND #4 AND PY=2015-2025 AND LA=(English)
```

**S10. Species combined with processing and extraction and limits**

```
#1 AND #5 AND PY=2015-2025 AND LA=(English)
```

**S11. Species combined with botanical factors and applications and limits**

```
#1 AND #6 AND PY=2015-2025 AND LA=(English)
```

**S12. Union of all Web of Science thematic searches**

```
#7 OR #8 OR #9 OR #10 OR #11
```

## Optional single-run Web of Science master query

```
TS=("Ilex paraguariensis" OR "I. paraguariensis" OR "yerba mate" OR "yerba maté" OR "erva
mate" OR "erva-mate" OR "Paraguay tea" OR "mate tea" OR "maté tea" OR chimarrao OR
chimarrão OR terere OR tereré) AND TS=((phytochem* OR "chemical composition" OR "chemical
profile" OR "chemical characterization" OR compositional OR polyphenol* OR phenolic* OR
"chlorogenic acid*" OR "caffeoylquinic acid*" OR flavonoid* OR methylxanthine* OR caffeine
OR theobromine OR saponin* OR mineral* OR nutrient* OR "proximate composition" OR "fatty
acid*" OR "organic acid*" OR sugar* OR metabolom* OR volatilom*) OR (pharmacolog* OR
bioactiv* OR antioxidant* OR "oxidative stress" OR anti-inflammatory OR inflamm* OR
antimicrobial* OR antibacter* OR antifung* OR antiviral* OR antidiabet* OR glycemic OR
glycaemic OR glucose OR insulin* OR hypolipid* OR lipid* OR cholesterol OR anti-obesity OR
antiobesity OR obes* OR "body weight" OR adipos* OR cardiovascular OR cardioprotect* OR
neuroprotect* OR cognit* OR anticancer OR antitumor OR antimutagen* OR cytotox* OR
clinical OR trial OR human* OR bioavailab* OR bioaccessib* OR pharmacokinetic* OR
microbiot* OR "glucagon-like peptide 1" OR "GLP-1") OR (safety OR toxic* OR "adverse
effect*" OR contaminant* OR "polycyclic aromatic hydrocarbon*" OR PAH OR PAHs OR
carcinogen* OR "cancer risk" OR "very hot" OR "beverage temperature" OR "caffeine
exposure" OR interaction* OR pregnancy OR lactation OR "heavy metal*" OR pesticide* OR
mycotoxin*) OR (extract* OR infusion* OR decoction* OR brew* OR solvent* OR "green
extraction" OR "ultrasound-assisted extraction" OR "ultrasound assisted extraction" OR UAE
OR "microwave-assisted extraction" OR "microwave assisted extraction" OR MAE OR
"supercritical fluid extraction" OR SFE OR "pressurized liquid extraction" OR PLE OR
"accelerated solvent extraction" OR ASE OR "natural deep eutectic solvent*" OR NADES OR
"deep eutectic solvent*" OR maceration OR Soxhlet OR drying OR roasting OR "smoke drying"
OR "smoke-drying" OR blanching OR sapeco OR storage OR encapsulat* OR formulat* OR "spray
drying" OR lyophiliz*) OR (leaf OR leaves OR stem* OR root* OR fruit* OR seed* OR residue*
OR byproduct* OR "by-product*" OR morpholog* OR anatom* OR genotype* OR cultivar* OR
cultivation OR agronom* OR "functional food*" OR nutraceutical* OR standardi* OR "quality
control" OR "food application*" OR "food product*" OR preservative* OR sensory OR
stability OR "shelf life" OR fermentation OR kombucha)) AND PY=2015-2025 AND LA=(English)
```

## Export, Reference Management, and Deduplication

All records should be exported with complete bibliographic metadata, abstracts, keywords, DOI, PMID or other database identifiers, and cited-reference information where available. Exports from all databases should be imported into EndNote and Zotero. Automated duplicate detection should be followed by manual verification using DOI/PMID, normalized title, first author, publication year, journal, volume, issue, and page or article number. Records that remain uncertain should be retained for title/abstract screening rather than removed automatically.

The number of records retrieved from each database, the number removed as duplicates, and the numbers screened, assessed in full text, excluded, and included must be taken from the actual exported library and screening log. The final manuscript and any flow diagram should use the same verified counts.
